# Strategies to Limit Benzodiazepine Use in Anesthesia for Older Adults: A Randomized Clinical Trial

**DOI:** 10.1001/jamanetworkopen.2024.42207

**Published:** 2024-10-31

**Authors:** Mark D. Neuman, Rui Feng, Aesha S. Shukla, Xiaoyan Han, Annamarie D. Horan, Karah Whatley, Marilyn M. Schapira, Edward R. Marcantonio, Richard P. Dutton

**Affiliations:** 1 Department of Anesthesiology and Critical Care, University of Pennsylvania Perelman School of Medicine, Philadelphia; 2 Center for Perioperative Outcomes Research and Transformation, University of Pennsylvania Perelman School of Medicine, Philadelphia; 3 Department of Biostatistics, Epidemiology, and Informatics, University of Pennsylvania Perelman School of Medicine, Philadelphia; 4 Center for Clinical Epidemiology and Biostatistics, University of Pennsylvania Perelman School of Medicine, Philadelphia; 5 US Anesthesia Partners, Dallas, Texas; 6 Department of Orthopaedic Surgery, University of Pennsylvania Perelman School of Medicine, Philadelphia; 7 Department of Medicine, University of Pennsylvania School of Medicine, Philadelphia; 8 Divisions of General Medicine and Gerontology, Department of Medicine, Beth Israel Deaconess Medical Center, Boston, Massachusetts; 9 Department of Medicine, Harvard Medical School, Boston, Massachusetts

## Abstract

**Importance:**

Despite guidelines recommending avoidance of benzodiazepine administration to older patients, many of them now receive benzodiazepines as a part of anesthesia care. The effectiveness of clinician- and patient-facing interventions to discourage such use remains insufficiently characterized.

**Objective:**

To evaluate the effect of clinician peer comparison, patient informational mail, or a combination of these interventions compared with usual care on the rate of perioperative benzodiazepine administration to older patients.

**Design, Setting, and Participants:**

This 2 × 2 factorial, stepped-wedge, cluster randomized clinical trial of a corporate quality improvement initiative was conducted between August 8, 2022, and May 28, 2023, across 415 hospitals, surgery centers, and physician offices in 8 US states served by anesthesia clinicians from a national anesthesia practice. Participants were adults aged 65 years or older who underwent an elective surgical or endoscopic procedure with general anesthesia. Data analyses followed the intention-to-treat principle.

**Intervention:**

Patients were randomly assigned to 1 of 4 groups—clinician peer comparison (wherein clinicians received feedback regarding their performance compared with other clinicians in the practice), patient informational mail (wherein patients received an informational letter encouraging them to have a discussion regarding medication selection with their clinician on the day of surgery), both interventions, or usual care (no intervention).

**Main Outcomes and Measures:**

Rate of benzodiazepine administration during anesthesia care and patient satisfaction with anesthesia care (measured by the Anesthesia Patient Satisfaction Questionnaire, version 2).

**Results:**

Among the 509 269 enrolled participants (255 871 females [50.2%]; mean [SD] age, 74 [7] years), 81 363 (16.0%) were assigned to clinician peer comparison, 98 520 (19.3%) to patient informational mail, 169 712 (33.3%) to both interventions, and 159 674 (31.4%) to usual care. Among patients who received benzodiazepine during anesthesia care, 24.5% were in the usual care group compared with 19.7% in the clinician peer comparison group, 20.0% in the patient informational mail group, and 19.7% in the combination group. After adjustment for time, none of the study interventions were associated with lower odds of benzodiazepine administration compared with usual care (odds ratio [OR], 1.02 [95% CI, 0.98-1.07]; *P* = .35 for clinician peer comparison; OR, 1.01 [95% CI, 0.96-1.05]; *P* = .81 for patient informational mail; and OR, 1.11 [95% CI, 1.05-1.16]; *P* < .001 for combined interventions). Satisfaction scores were high in all groups and did not vary by treatment assignment.

**Conclusions and Relevance:**

This randomized clinical trial found that clinician peer comparison, patient informational mail, or a combination of both interventions did not reduce benzodiazepine administration to older patients compared with usual care; patient satisfaction remained high throughout the study. Overall, the findings suggest a need to explore other patient-targeted interventions to improve anesthesia care.

**Trial Registration:**

Clinicaltrials.gov Identifier: NCT05436392

## Key Points

**Question:**

Does clinician peer comparison, patient informational mail, or a combination of both reduce benzodiazepine administration in anesthesia to older patients compared with usual care?

**Findings:**

In this randomized clinical trial involving 509 269 older adults who underwent a surgical procedure, the odds of benzodiazepine administration were not significantly lower among patients assigned to any of the 3 interventions—clinician peer comparison, patient informational mail, or both—compared with usual care received before surgery. Patient satisfaction with anesthesia care was high throughout the study period.

**Meaning:**

Findings of this trial indicate the need to examine other patient-targeted interventions to improve anesthesia care.

## Teaser

This randomized clinical trial examines the efficacy of 3 clinician- and patient-facing strategies to reduce the use of benzodiazepines in anesthesia among older adults undergoing surgery.

## Introduction

Over 21 million surgical procedures are performed annually for US adults aged 65 years or older.^1,2^ Over half of these individuals receive a benzodiazepine, such as midazolam, during anesthesia care,^3^ typically with the intent of improving patient experience.^4,5^ Yet randomized clinical trials have demonstrated that benzodiazepines do not improve satisfaction with anesthesia compared with placebo.^6,7^ Current guidelines recommend against administering benzodiazepines to older patients due to potential adverse effects.^8,9^

Past studies on clinician-facing behavioral strategies to discourage benzodiazepine administration to older adults during anesthesia care used single-center, nonrandomized designs.^10,11^ Moreover, no study has examined patient-facing interventions. Generalizable evidence on effective patient and clinician interventions for reducing benzodiazepine administration to older patients is needed to inform quality improvement efforts.

We conducted a randomized clinical trial of a corporate quality improvement initiative (De-adopting Routine Preoperative Benzodiazepines for Older Surgical Patients [DROP-Benzo]) for a private medical group. The objective of this trial was to evaluate the effect of clinician peer comparison, patient informational mail, or a combination of these interventions compared with usual care on the rate of perioperative benzodiazepine administration to older patients. We hypothesized that patients receiving a study intervention would be less likely to receive a benzodiazepine during anesthesia care vs usual care.

## Methods

### Design, Oversight, and Population

DROP-Benzo, a 2 × 2 factorial, stepped-wedge cluster randomized clinical trial, was conducted in 22 divisions of a national anesthesia private practice (US Anesthesia Partners) between August 8, 2022, and May 28, 2023. The University of Pennsylvania Institutional Review Board approved the trial protocol and waived the informed consent requirement because the study posed no more than minimal risk to participants and could not be carried out practicably without such a waiver and the waiver was judged to not adversely affect the rights or welfare of the participants. The protocol and statistical analysis plan are provided in Supplement 1. The analysis plan was finalized prior to initiating data analysis. We followed the Consolidated Standards of Reporting Trials (CONSORT) reporting guideline.

During the study period, approximately 4200 anesthesiologists, certified registered nurse anesthetists (CRNAs), and certified anesthesiologist assistants across 22 practice divisions provided contracted anesthesia services to 415 hospitals, surgery centers, and physician offices (care locations) in 8 states (eTable 1 in Supplement 2). We planned the trial with 24 divisions encompassing 423 care locations; however, 2 divisions (8 care locations) left the practice before the start date and were not included in the study.

Patients were included if they were aged 65 years or older and undergoing a scheduled surgical or endoscopic procedure with general anesthesia; we excluded urgent or emergent cases because we could not reliably deliver the patient-facing interventions to this group. Additionally, we excluded patients who received a nerve block or who exclusively received monitored anesthesia since benzodiazepines may be appropriate for periprocedural sedation in these cases.

### Randomization

**Figure 1.**
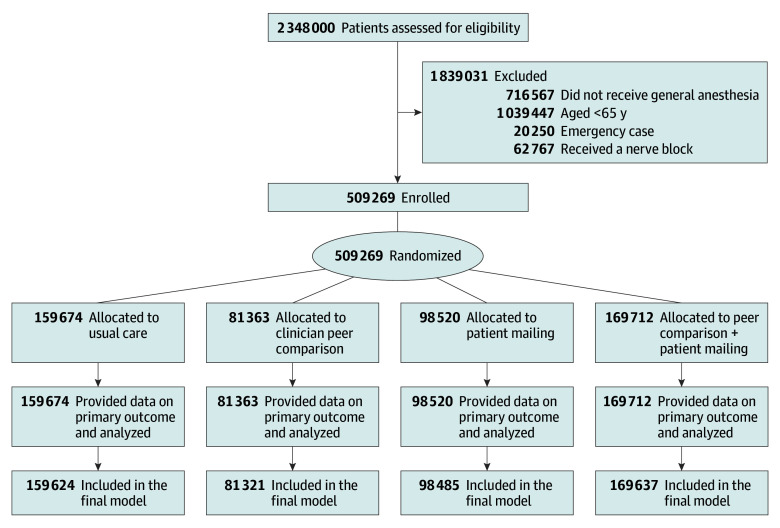
Patient Screening, Enrollment, and Follow-Up

We randomly assigned patients in the 22 divisions to 1 of 4 treatment sequences (ie, 6 divisions per sequence) (Figure 1). Each sequence comprised a unique 5-period intervention implementation scheme (eFigure 1 in Supplement 2), resulting in 110 division-period clusters. Sequence assignments were made with stratified randomization across 6 strata comprising 4 divisions each using the fixed-block randomization algorithm. We defined the strata based on prior-year eligible case volumes within each division. At the time of study planning, the partner organization was in the process of establishing electronic medical record (EMR) data access for quality monitoring within 4 divisions. We assigned these divisions to a single stratum to permit future analyses of EMR data if these data were to become available.

At the start of the trial, the study statistician (R.F.) communicated each division’s assigned treatment sequence via email to investigators at the partner organization (R.P.D. and A.S.S.), who then oversaw implementation of study procedures according to this assignment. We maintained the randomization algorithm, code, and original sequence on a secure server, which were accessible to the study data management team (R.F. and X.H.) and unknown to the principal investigator (M.D.N.) and remaining study personnel. We did not mask clinicians or patients to treatment assignment.

In the initial 10 weeks, no divisions received either intervention (clinician peer comparison or patient informational mail). We had initially planned an 8-week duration for this initial period, but an additional 2 weeks were permitted at the request of the partner organization to accommodate scheduling conflicts. Each intervention (peer comparison feedback or the patient informational mail) was subsequently initiated at a new group of practices at 8-week intervals. We rolled out interventions in an overlapping fashion (eFigure 1 in Supplement 2) such that each sequence had at least 1 period in which the assigned divisions received only 1 of the study interventions prior to adding the second intervention. Involved clinicians were notified of the project by email from practice leaders at the beginning of the trial, and relevant practice guidelines^8,9^ were made available via the practice website. At 63 care locations across 8 divisions, clinicians used electronic anesthesia record systems that allowed the quality management group at the practice to directly track benzodiazepine administration. At the remaining 352 care locations, benzodiazepine administration was tracked via required electronic self-reporting through the practice’s electronic billing and quality management platform, which was introduced between May and July 2022.

### Treatment Arms or Interventions

#### Clinician Peer Comparison

Anesthesiologists, CRNAs, and anesthesiologist assistants at care locations received monthly individual reports via their professional email address from corporate accounts, which were regularly used to communicate practicewide updates. These reports showed the clinician’s fraction of general anesthesia cases for patients aged 65 years or older to whom the clinician administered a benzodiazepine during anesthesia care. Each clinician was compared to all other clinicians in the practice to establish a uniform standard for improvement across the practice. Comparisons used a 3-month rolling mean of data from the practice’s electronic billing and quality management platform using the electronic anesthesia record system and clinician attestation. Following the recommendations for design of clinician peer comparison,^12,13,14^ we compared clinicians with performance below the group median scores to the median percentile of performance, whereas clinicians above this level were compared with the 10th percentile of performance. Clinicians at or above the 10th percentile were notified of their scores and encouraged to continue high performance (eFigures 2-4 in Supplement 2).

#### Patient Informational Mail

We designed electronic patient mail with input from researchers, practice leaders, and community members. Based on this input, the intervention consisted of an informational letter encouraging patients to engage in discussions with their anesthesia clinicians on the day of surgery regarding medication selection (eFigure 5 in Supplement 2). In units of patients assigned to receive this intervention, the mail was distributed via email 2 weeks prior to surgery, printed on corporate letterhead, and was signed by the practice chief quality officer (R.P.D.). Mail was available in both English and Spanish formats. We assessed patient engagement by the percentage of mail opened out of all the emails sent at 4 practice divisions.

### Primary and Safety Outcomes

The primary outcome was a binary variable indicating benzodiazepine administration by an anesthesia clinician at any time during anesthesia care. Outcome data were collected via an electronic anesthesia record system and clinician attestation. For patients undergoing general anesthesia, benzodiazepines are most often used as premedication prior to anesthesia induction; thus, we assumed this outcome would reflect preoperative benzodiazepine use. However, we did not explicitly distinguish between preinduction administration vs administration at other times during anesthesia care.

We measured patient satisfaction using 8 items from the Anesthesia Patient Satisfaction Questionnaire, version 2 (APSQ-2).^15^ These 8 items rate overall anesthesia experience and aspects of interactions with the anesthesia team on a scale of 1 to 5 (score range: 1-5, with higher scores indicating greater satisfaction). We dichotomized responses for analysis into favorable vs neutral or unfavorable based on selection of either of the 2 highest categories for each item. Satisfaction information was collected using an English or Spanish web-based APSQ-2 issued by text or email 3 days after surgery, with up to 3 weekly completion reminders sent to nonrespondents.

We collected from anesthesia records additional data on principal surgical procedure type, age, sex, American Society of Anesthesiologists (ASA) Physical Status classification (score range: I [indicating no systemic disease] to IV or V [indicating severe systemic disease that is a constant threat to life or moribund patient]), insurance type, care team structure, service location, and clinician tenure with the study practice. Since race and ethnicity are not captured in the anesthesia records, we collected this information directly from participants using a web-based questionnaire, which allowed us to conduct preplanned subgroup analyses by race and ethnicity. Race categories were American Indian or Alaska Native, Asian, Black or African American, Native Hawaiian or Other Pacific Islander, White, and Other. Ethnicity categories were Hispanic or Latino and not Hispanic or Latino. For analysis, Native Hawaiian or Other Pacific Islander was grouped with the Other category due to low numbers. Individuals reporting more than 1 race were assigned to a separate category.

Baseline data were compared across groups using standardized differences. We considered standardized differences greater than 0.2 to be an important imbalance.

Clinicians reported safety outcomes in the practice’s uniform electronic adverse event reporting system. The principal investigator (M.D.N.) grouped reported events into 12 categories based on organ system and event type (cardiac, respiratory or airway, neurological, vascular, bleeding or transfusion, pain, nausea or vomiting, hyperthermia or hypothermia, hyperglycemia or hypoglycemia, procedural complications and medication errors, death, and other [eg, case cancellation, equipment malfunction, need to return to operating room, and need for intensive care unit admission]). Due to unavailability of EMR data for most patients in the trial, we were unable to analyze EMR data on safety outcomes.

### Statistical Analysis

We anticipated a total enrollment of approximately 225 000 eligible patients. We estimated a baseline benzodiazepine use rate of 45% based on prior research^3^ and available practice data. We projected that this sample would provide over 90% power for a 2–percentage point change in the primary outcome for each intervention alone, and a 4–percentage point change for the combined intervention, at a Bonferroni-adjusted significance of *P* = .02 and an intracenter correlation coefficient of 0.3.^16^

Analyses followed the intention-to-treat principle, wherein we analyzed data on all participants according to the treatment assigned via cluster randomization. We used mixed-effects logistic regression to estimate the treatment effect of clinician peer comparison, patient informational mail, or the combined intervention vs usual care (control) on the odds of receiving a benzodiazepine during anesthesia care. This model contained binary indicator variables for the treatment status of each unit within a given period for each of the 2 interventions and an interaction term capturing the joint effect of the interventions. The model included 2-level random effects, with 1 random effect for sequence assignment and the other for division nested within each sequence. To increase the precision of the treatment estimate, the model was adjusted for ASA Physical Status classification (III or higher [indicating severe disease] vs I or II [indicating no disease to mild disease]), surgical procedure type, age, sex, and study period by including them as fixed effects^17,18,19^ (eMethods in Supplement 2). Race and ethnicity were not included in the logistic model due to a high rate of missing data. Models addressed clustering via random effects.

We assessed for heterogeneity of treatment effects on the primary outcome by adding interaction terms to the main study model for (1) age (≥85 years vs ≤84 years); (2) sex, (3) race (Black, White, or other); (4) ethnicity (Hispanic vs non-Hispanic); (5) insurance type; (6) surgical procedure type; (7) whether the division fell above or below the practicewide 50th percentile of benzodiazepine administration; (8) clinician tenure in the practice (above or below the practicewide 50th percentile); (9) care model (solo anesthesiologist vs anesthesiologist-CRNA care team); and (10) service location (inpatient hospital or emergency department vs ambulatory surgery center, physician office, or hospital-based outpatient facility).

We used descriptive statistics to characterize patient satisfaction scores and adverse events across study groups. We compared rates of missing data across divisions and treatment arms. Study models used complete data; we planned additional sensitivity analyses if primary outcome data were missing in 10% or more of cases. Analyses used SAS 9.4 (SAS Institute Inc).

## Results

We enrolled 509 269 patients (255 871 females [50.2%], 253 398 males [49.8%]; mean [SD] age, 74 [7] years). Of these patients, 31.4% (159 674) received usual care, 16.0% (81 363) received the clinician peer comparison, 19.3% (98 520) received the patient informational mail, and 33.3% (169 712) received both interventions (Figure 1). At divisions where engagement with the patient informational mail was tracked, 42.0% of patients accessed the intervention.

Patient and surgery characteristics were similar across treatment arms (Table 1). Mean (SD) case duration was between 95 (82) minutes and 100 (86) minutes in each group. Distribution of ASA Physical Status classification, surgical procedure type, insurance type, clinician tenure in the study practice, care model, and service location were also similar across groups. Information on race and ethnicity was available for approximately 5% of all enrolled patients (eTable 2 in Supplement 2). Standardized differences were less than 0.2 for all covariates (eTable 3 in Supplement 2).

**Table 1.** Characteristics of the Study Population

Characteristic	Participants, No. (%)			
	Usual care	Clinician peer comparison	Patient informational mail	Clinician peer comparison + patient informational mail
Age, mean (SD), y	74 (7)	74 (7)	74 (7)	74 (7)
Sex				
Male	79 174 (49.6)	40 929 (50.3)	48 476 (49.2)	84 819 (50.0)
Female	80 500 (50.4)	40 434 (49.7)	50 044 (50.8)	84 893 (50.0)
Case duration, mean (SD), min	100 (86)	101 (84)	95 (82)	100 (85)
ASA Physical Status classification				
I: No systemic disease	1428 (0.9)	763 (0.9)	752 (0.8)	1285 (0.8)
II: Mild systemic disease	45 099 (28.3)	21 751 (26.8)	27 676 (28.1)	44 051 (26.0)
III: Severe systemic disease	92 785 (58.1)	47432 (58.3)	58 714 (59.6)	10 1782 (60.0)
IV or V: Severe systemic disease that is a constant threat to life or moribund patient	20 312 (12.7)	11 375 (14.1)	11 343 (11.5)	22 519 (13.3)
Surgical procedure type				
Endoscopic	31 378 (19.7)	15 024 (18.5)	20 334 (20.6)	31 873 (18.8)
General or abdominal	25 869 (16.2)	12 261 (15.1)	14 529 (14.7)	26 417 (15.6)
Orthopedic	22 167 (13.9)	12 757 (15.7)	15 279 (15.5)	24 829 (14.6)
Urologic	19 147 (12.0)	9370 (11.5)	8843 (9.0)	19 834 (11.7)
Neurosurgery or spine	17 923 (11.2)	8486 (10.4)	12 435 (12.6)	18 402 (10.8)
Cardiologic	9575 (6.0)	5661 (7.0)	6640 (6.7)	10 462 (6.2)
Vascular	5644 (3.5)	3135 (3.9)	3446 (3.5)	6211 (3.7)
Cardiac	4931 (3.1)	2740 (3.4)	3190 (3.2)	5213 (3.1)
ENT	4406 (2.76)	2164 (2.66)	2211 (2.2)	4573 (2.7)
Gynecologic	4285 (2.7)	1714 (2.1)	2597 (2.6)	4490 (2.6)
Thoracic	3894 (2.4)	2123 (2.6)	2198 (2.2)	4652 (2.7)
Ophthalmologic	3221 (2.0)	1784 (2.2)	2015 (2.0)	3072 (1.8)
Cosmetic	2970 (1.9)	1372 (1.7)	1267 (1.3)	2825 (1.7)
Radiologic	2177 (1.4)	1175 (1.4)	1546 (1.6)	2506 (1.5)
Other^a^	2087 (1.3)	1597 (2.0)	1990 (2.0)	4353 (2.6)
Insurance type				
Commercial	13 622 (8.5)	7020 (8.6)	9144 (9.3)	15 125 (8.9)
Governmental	143 373 (89.8)	73 029 (89.8)	88 072 (89.4)	15 2084 (89.6)
Other^b^	2679 (1.7)	1314 (1.6)	1304 (1.3)	2503 (1.5)
Clinician tenure in practice, median (IQR), y	9.6 (4.1-17.5)	9.6 (4.0-18.1)	10.0 (4.9-18.1)	9.0 (2.8-17.4)
Care model				
Solo	56 121 (35.3)	31 824 (39.3)	35 592 (36.3)	48 286 (28.8)
Team	103 000 (64.7)	49 075 (60.7)	62 423 (63.7)	119 567 (71.2)
Service location				
Inpatient hospital or ED	57 020 (35.7)	32 257 (39.6)	32 560 (33.0)	61 485 (36.2)
Other^c^	102 654 (64.3)	49 106 (60.4)	65 960 (67.0)	108 227 (63.8)

Abbreviations: ASA, American Society of Anesthesiologists; ED, emergency department; ENT, ear, nose, and throat.

^a^ Other surgical procedure type includes mom/baby, cardiovascular, psychiatry, radiation oncology, and not otherwise specified.

^b^ Other insurance type includes self-pay, charity care, and not otherwise specified.

^c^ Other service location includes ambulatory surgery center, physician office, and hospital-based outpatient department.

**Figure 2.**
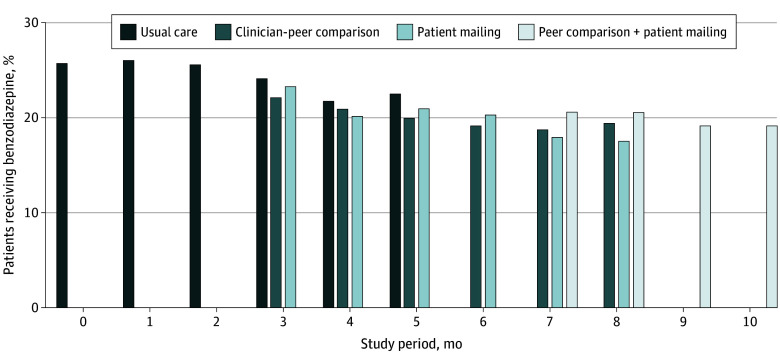
Benzodiazepine Administration Over the Study Period by Month and Treatment Arm The percentage of patients receiving benzodiazepine decreased over time in each treatment arm.

Primary outcome data were available for all enrolled patients, and 0 patients were lost to follow-up. Among patients assigned to usual care, 24.5% received a benzodiazepine during anesthesia care compared with 19.7% assigned to clinician peer comparison, 20.0% assigned to patient informational mail, and 19.7% assigned to both interventions (*P* < .001). Benzodiazepine administration declined over the course of the study in all arms (Figure 2).

The logistic mixed-effects model included 509 067 patients, 99.9% of all enrolled. Of these patients, 202 (0.04%) had missing data on ASA Physical Status classification and were not included in the model; data were complete for all other patients. After adjustment for study period and other covariates, none of the interventions compared with usual care was associated with lower odds of benzodiazepine administration (odds ratio [OR], 1.02 [95% CI, 0.98-1.07]; *P* = .35 for clinician peer comparison; OR, 1.01 [95% CI, 0.96-1.05]; *P* = .81 for patient informational mail; and OR, 1.11 [95% CI, 1.05-1.16]; *P* < .001 for the combined intervention) (Table 2). We obtained similar results with adjustment for study period only, without other covariates (eTable 4 in Supplement 2).

**Table 2.** Primary Outcome Analyses

Group	Received Intervention?		No. of patients	Patients receiving a benzodiazepine, No. (%)	OR (95% CI)^a^
	Clinician peer comparison	Patient informational mail			
Usual care	No	No	159 674	39 191 (24.5)	1 [Reference]
Clinician peer comparison	Yes	No	81 363	16 051 (19.7)	1.02 (0.98-1.07)
Patient informational mail	No	Yes	98 520	19 677 (20.0)	1.01 (0.96-1.05)
Clinician peer comparison + patient informational mail	Yes	Yes	169 712	33 373 (19.7)	1.11 (1.05-1.16)

Abbreviation: OR, odds ratio.

^a^ Models adjusted for study period, American Society of Anesthesiologists Physical Status classification, surgery type, age, and sex.

Subgroup analyses suggested a small decrease in benzodiazepine administration with the patient informational mail vs usual care among White patients (OR, 0.74; 95% CI, 0.56-0.92; *P* = .007), non-Hispanic or Latino patients (OR, 0.76; 95% CI, 0.62-0.95; *P* = .01), and patients who underwent otolaryngological or ophthalmological procedures (OR, 0.84; 95% CI, 0.72-0.97; *P* = .02). We did not observe decreases in benzodiazepine administration with the study interventions across other subgroups (eFigure 6 in Supplement 2).

**Figure 3.**
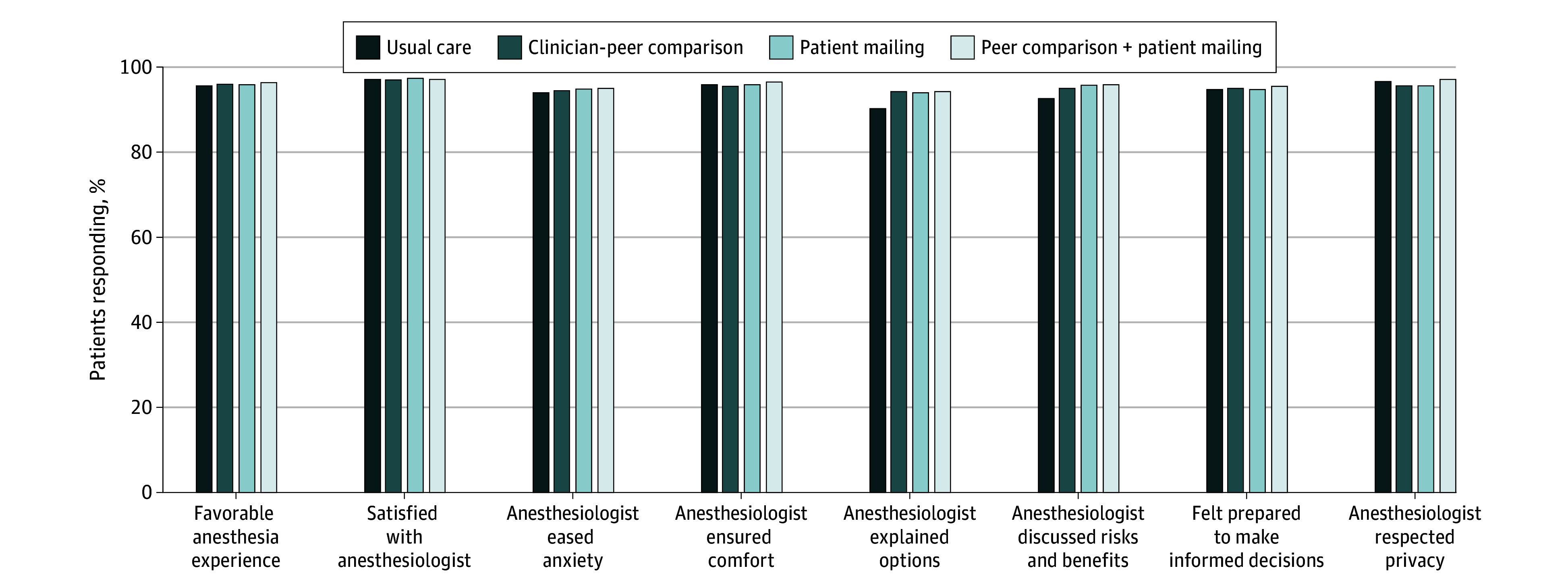
Patient Satisfaction With 8 Domains of Anesthesia Care Individual questionnaire items, coding, and response data are provided in eTable 5 in Supplement 2. Denominators for each item ranged from 29 389 (discussed risks and benefits) to 38 705 (favorable anesthesia experience) due to nonresponse. Anesthesia Patient Satisfaction Questionnaire, version 2 was used for assessment (score range: 1-5, with higher scores indicating greater satisfaction).

A total of 39 326 patients (7.7%) answered at least 1 item on the postoperative satisfaction survey (APSQ-2). Among those with available information, patient satisfaction with anesthesia care did not differ across intervention groups and was favorable throughout the study period. A score of 4 or more was reported by at least 90% of patients across all items (Figure 3; eTable 5 in Supplement 2). Adverse events did not vary across treatment arms (eTable 6 in Supplement 2).

## Discussion

Among the 509 269 older adults included in the DROP-Benzo trial, clinician peer comparison, patient informational mail, or a combination of both did not reduce benzodiazepine administration compared with usual care. Patient satisfaction with anesthesia care was similar across groups and high overall.

Clinician peer comparison has previously been shown to result in guideline adherence in primary care^14^ and acute care settings.^20,21^ In the context of anesthesia, clinician peer comparison may improve adherence to recommended ventilator management strategies during general anesthesia care.^22^ Results of the present trial contrast with these findings and other evidence of the effectiveness of clinician peer comparison.^21^ This discrepancy suggests that clinician peer comparison may be less effective in discouraging benzodiazepine administration during anesthesia care than other aspects of care. A previous study found that introduction of an EMR-based best practice alert was associated with a decrease in benzodiazepine administration^10^; thus, the present findings may argue that clinician-facing interventions targeting benzodiazepine administration during anesthesia care may vary in their effectiveness depending on their level of integration into clinical workflows.^23^

In nonsurgical settings, patient empowerment interventions may be effective for promoting deprescribing of potentially inappropriate medications in older adults.^24,25^ We designed the patient informational mail with input from community members and clinical leaders; however, the simple mail received by patients may have been insufficient to alter benzodiazepine administration patterns. It is also possible that benzodiazepine administration during anesthesia care may be less amenable to patient empowerment interventions than other aspects of care.

We observed a reduction in benzodiazepine administration over the study period across all groups. This result may have been due to the practicewide email notification issued at the start of the trial or may have been an unintended consequence of the required self-reporting by clinicians of benzodiazepine administration within some divisions. Although we introduced this requirement 3 months prior to the start of the trial, it is possible that allowing a longer interval may have permitted greater washout of any effect on care patterns of required self-reporting of benzodiazepine administration. While unadjusted benzodiazepine administration rates were lower in each of the 3 intervention groups than in usual care, these differences did not persist after adjusting for the overall reduction in administration over time. Although benzodiazepine administration decreased over time, patient satisfaction with anesthesia care remained high throughout the study period, confirming past trial evidence that avoidance of benzodiazepines does not diminish patient satisfaction.^6,7^

Considered as a whole, trial findings suggest that clinician peer comparisons do not add a benefit to basic efforts to discourage routine benzodiazepine administration during anesthesia care, such as routine measurement of use and prioritization by practice leaders. Similarly, simple patient informational mail may have limited impact on benzodiazepine administration; the potential for other patient-targeted interventions to improve anesthesia care should be explored in future work. Additionally, the lack of a change in patient satisfaction confirms evidence from prior trials^6,7^ that reducing benzodiazepine administration during anesthesia care does not adversely affect satisfaction.

### Limitations

Advanced notification of clinicians regarding the intervention and practicewide data collection for the primary outcome may have introduced change in the practice independent of the study interventions. However, these design features were required by the standard operating procedures of the practice and to permit data collection across multiple EMRs and electronic anesthesia record systems used by participating clinicians. Additionally, the analytic approach we used allowed us to avoid spurious findings by accounting for time in the study models. It is possible that we may have obtained a different result had we allowed a longer interval between the introduction of clinician attestations and the start of data collection. Given these findings, we anticipate that even under such conditions, any impact of the interventions on practice would be small. At care locations wherein we measured engagement with the patient-facing intervention, participants opened the informational mail at a rate comparable to that reported in other studies.^26,27^ However, it is possible that increasing patient engagement with this intervention could produce different results.

## Conclusions

In this DROP-Benzo randomized clinical trial of 3 corporate quality improvement interventions (clinician peer comparison, patient informational mail, and a combination of both), the interventions did not reduce benzodiazepine administration to older patients compared with usual care. Patient satisfaction with anesthesia care was high overall across treatment groups. There is a need to explore other patient-targeted interventions to improve anesthesia care.

## Notes

Supplement 1.
Trial Protocol

Supplement 2. **eTable 1.** Study Divisions **eFigure 1.** Study Randomization Scheme **eFigure 2.** Physician Peer Comparison Message: High-Performer Alert **eFigure 3.** Physician Peer Comparison Message: Moderate-Performer Alert **eFigure 4.** Physician Peer Comparison Message: Low-Performer Alert **eFigure 5.** Patient Informational Mailing Text **eTable 2.** Race and Ethnicity Information for Study Participants Providing Data **eTable 3.** Standardized Differences for Individual Study Covariates Versus Control Group **eMethods.** Information on Mixed-Effects Logistic Model **eTable 4.** Simplified Regression Model Results for Primary Outcome **eFigure 6.** Subgroup Analyses **eTable 5.** Patient Satisfaction Item Responses **eTable 6.** Adverse Events by Study Group

Supplement 3.
Data Sharing Statement
