# Supplementary material for: Strategies to Limit Benzodiazepine Use in Anesthesia for Older Adults: A Randomized Clinical Trial
Source: JAMA Netw Open. 2024 Oct 31;7(10):e2442207. doi: 10.1001/jamanetworkopen.2024.42207 (PMC11528310; doi:10.1001/jamanetworkopen.2024.42207)
Supplement: Supplement 1. — Trial Protocol [file jamanetwopen-e2442207-s001.pdf]

Supplement to: Neuman MD, Feng R, Shukla A, et al. Strategies to limit benzodiazepine use in anesthesia for older surgical patients: a randomized clinical trial

This supplement contains the following items:

A. Initial approved study protocol (version date: January 31, 2022)

B. Final study protocol (version date: June 26, 2022)

C. Summary of protocol changes

D. Statistical analysis plan (version date: August 1, 2023)

DROP-Benzo (De-adopting Routine Preoperative Benzodiazepines for Older Surgical Patients): a randomized trial of behavioral strategies to reduce unnecessary midazolam administration to older surgical patients

Study Protocol

January 31, 2022

Outline

1. Abstract
2. Overall objectives
3. Aims
    3.1 Primary outcome
    3.2 Secondary outcomes
4. Background
5. Study design
    5.1 Design
    5.2 Study duration
    5.3 Target population
    5.4 Accrual
    5.5 Key inclusion criteria
    5.6 Key exclusion criteria
6. Subject recruitment
7. Subject compensation
8. Study procedures
    8.1 Consent
    8.2 Procedures
9. Analysis plan
10. Human research protection
    10.1 Data confidentiality
    10.2 Subject confidentiality
    10.3 Subject privacy
    10.4 Data disclosure
    10.5 Data safety and monitoring
    10.6 Risk/benefit
        10.6.1 Potential study risks
        10.6.2 Potential study benefits
        10.6.3 Risk/benefit assessment

1. Abstract

Over 21 million surgical procedures take place among adults aged 65 and older in the US each year, and most older surgical patients in the US now receive benzodiazepines (e.g., midazolam, lorazepam) during anesthesia care. This occurs despite recommendations to avoid these medications in older patients due to associated medical risks and lack of demonstrated benefit. In other words, routine benzodiazepine administration to older surgical patients is likely to represent low-value care that is a suitable target for de-adoption. In this study, we will evaluate a United States Anesthesia Partners (USAP, Dallas, TX) quality improvement initiative using peer comparison feedback to clinicians and/or mailed informational letters to patients as strategies to encourage physician de-adoption of routine preoperative benzodiazepine administration to older surgical patients. In partnership with USAP, this study will be conducted using randomization to evaluate its effect.

2. Overall objectives

The objective of this research study is to evaluate the effect of a corporate quality improvement initiative carried out by a private medical group practice aiming to change clinician preoperative benzodiazepine administration behaviors for older adults using clinician peer comparison feedback and mailed informational letters to patients.

3. Aims

3.1 Primary outcome

The primary outcome measure is the change in the rate of benzodiazepine administration among eligible surgical cases.

3.2 Secondary outcome

The secondary outcome measure is the change in patient satisfaction with care, as measured by Anesthesiologist Patient Satisfaction Questionnaire Composite Satisfaction Score, Version 2 (APSQ2).

3.3 Exploratory outcomes

We will explore several other outcomes of interest. These will include changes in dose and type of administered benzodiazepines; selected APSQ2 composite domain scores; 30-day delirium and pulmonary complications; and hospital length of stay for patients not discharged directly home after surgery.

4. Background

Benzodiazepines (e.g., midazolam, lorazepam) are sedative-hypnotic medications[2] that are frequently administered during surgical anesthesia care. Over 21 million major surgeries occur each year among US adults aged 65 and older,[3,4] and available data[5,6] indicate that over half of all older surgical patients now receive benzodiazepines during anesthesia care. For example, in a recent study of 24,683 patients aged 65 and older undergoing a range of non-cardiac surgeries at 4 hospitals in one US academic health system from 2014-2018, we found that 64.5% (95% CI: 64.0%, 64.9%) received a benzodiazepine during anesthesia; while the rate of benzodiazepine administration decreased with increasing age, 23.8% (95% CI: 21.8%, 25.8%) of individuals aged 85 and older still received a benzodiazepine.[7]

For patients undergoing general anesthesia (medically induced unconsciousness), benzodiazepines are most commonly administered as an adjunctive therapy delivered immediately before the induction of

anesthesia with the intent of reducing anxiety and improving patient experience. Nonetheless, a 2015 multicenter randomized study of 1,062 patients found comparable satisfaction scores after surgery among patients assigned to receive a benzodiazepine, placebo, or no premedication, with higher rates of residual sedation after surgery in the benzodiazepine group.[8] Similar findings were obtained in a subsequent observational study of 260 patients who received a benzodiazepine versus no premedication before general anesthesia.[9] Benzodiazepines carry dose-dependent risks for older surgical patients, including delirium[10-15] and respiratory depression.[16] Taken together, available evidence suggests that much benzodiazepine administration to older adults undergoing general anesthesia is unlikely to produce benefits and may cause harm; in other words, it represents low-value care[17,18] that is suitable for de-adoption.[19,20] Indeed, the American Geriatrics Society[21,22] and others[23] recommend that benzodiazepines be avoided in older surgical patients.

In this context, persistently high rates of benzodiazepine administration to older surgical patients nationally highlight a need for effective, scalable interventions to support de-adoption of this low-value practice. Behavioral economic nudges represent promising but largely unexplored strategies to reduce excessive benzodiazepine administration to surgical patients. "Nudges" are subtle changes to the framing of information that can influence behavior.[24,25] Clinician-facing nudges that incorporate peer comparisons can improve medication selection and prescribing[26-28] by countering common biases and heuristics[29] that can contribute to failures to appropriately tailor medication regimens based on individual patient characteristics.[30] Patient-facing nudges, such as simple informational letters represent an additional strategy to motivate practice change. In other contexts, simple informational letters have been shown to activating patients to participate in medical decisions.[31-33] In the present context, simple informational letters from practice leaders may prompt patients to initiate conversations with providers regarding care at the time of surgery, potentially influencing provider treatment decisions.

5. Study design

5.1 Design

This research study will analyze results from a USAP initiative that uses a four-arm factorial stepped-wedge cluster randomized trial. Both interventions (clinician peer comparisons and informational patient letters) will be concurrently rolled out across 24 physician practice divisions within USAP. Timing of intervention roll-out within each division will be randomly assigned to permit evaluation of the effect of each intervention individually and combined. The total intervention period will be approximately 9 months (40 weeks) in duration.

5.2 Study duration

The study is expected to take 2 years to conduct including planning (6 mo), the 9-month active phase of trial, a 1-month period for final data collection, and 8 months for analysis and dissemination of results.

5.3 Target populations

Anesthesia clinicians employed by USAP administering general anesthesia to patients aged 65 and older.

5.4 Accrual

This is an evaluation of a health system intervention of approximately 4,200 clinicians across 24 USAP physician practice divisions.

5.5 Key inclusion criteria

All anesthesia clinicians employed by USAP will be included in the evaluation.

In the research study analysis, patients will be included in the primary sample if they are at least aged 65 and undergo an elective (scheduled) surgical or endoscopic procedure under general anesthesia.

5.6 Key exclusion criteria

In the research study analysis, patients will be excluded if they are unscheduled (urgent/emergent) cases due to inability to reliably deliver study interventions to this group; if they did not receive general anesthesia; or if they received a nerve block procedure.

6. Subject recruitment

Since this is an evaluation of a corporate quality improvement intervention, clinicians and patients will not be recruited or enrolled individually but instead an analysis will be conducted based on anesthesia cases. We estimate that the sample will include approximately 4,200 clinicians and 225,000 patients.

7. Subject compensation

No compensation will be offered in this study.

8. Study procedures

8.1 Consent

A waiver of informed consent is requested from both clinicians and patients. This is a corporate quality improvement initiative that will be implemented with or without the proposed research study. The study is an evaluation of that implementation which has support from leadership at USAP. Therefore, clinicians and their patients will not be consented as this is the standard of practice within the context of the corporate quality improvement initiative. Without a waiver of the consent, the initiative would still be implemented by the health system, but the study would be infeasible. There are several additional reasons why we feel a waiver of consent should be granted. First, it is not feasible to consent every clinician and patient at the 24 practice divisions, which span 8 US states and serve hundreds of procedural care locations. Second, if clinicians or patients in the control group were consented, they would know they were being monitored and this could influence their behavior. This could potentially disrupt the design of the evaluation and make interpretation of the findings challenging. Third, clinicians are not being forced to administer or avoid benzodiazepines in a specific manner, and patients are not being forced to request or decline benzodiazepines. Instead, they are each being guided toward evidence-based care practices but maintain their ability to act as they feel is appropriate. This is no different than standard of care in which clinicians and patients each make decisions based on available information. Since the study will not involve collection of information whose provision would directly impacts on the safety or welfare of subjects, we do not plan to undertake additional communication with enrolled patients to provide additional pertinent information regarding their participation or study findings.

8.2 Procedures

Randomization procedures. The following procedure will be used at USAP to randomize divisions, informed by study team input. Each division will be a randomization unit. The divisions that will contribute data on complications and length of stay will be in the same block. The remaining divisions will be separated into 5 other blocks of 4 divisions each to obtain similar characteristics within each block, guided by the k-means clustering algorithm. Within each block, each division will be randomized into one of the 4 predefined treatment sequences (**Figure**) using the fixed block randomization algorithm.

In the initial 8-week period of the study, no clusters will receive either intervention. During this baseline data collection phase, all USAP clinicians will be receive standard organization educational materials regarding preoperative medication use in older surgical patients, incorporating available best evidence on potentially avoidable medications in the elderly. Providers will receive communication during this phase notifying them of the quality improvement initiative and its components. Subsequently, clusters will be randomized at 8 week intervals to receive either peer comparison feedback or the patient informational letter, followed by a period were both interventions are simultaneously deployed at all divisions. The USAP Chief Quality Officer will receive notification of each site's randomization status two weeks before the beginning of each step in the randomization sequence and oversee implementation of the below study procedures according to the randomization assignment. The randomization algorithm, code, and original sequence will be maintained on a secure server, accessible by the study data management team, and blinded to other study personnel.

Peer comparison feedback will use data on the number of eligible cases in which a benzodiazepine was administered. Providers will receive monthly alerts via USAP's smartphone-based practice management application presenting individual-level data on their benzodiazepine administration patterns versus other USAP providers, using data from the USAP clinical and quality data warehouse. Comparative data will be delivered using a 3-month rolling average as follows: (a) If clinician is above median: informed how their data compares to the median; (b) If clinician is below median but above 10th percentile: informed how their data compares to the 10th percentile; (c) If clinician is 10th percentile or below: informed of their data and commended for being a "high performer." Data will be compared to peer clinicians. Alert deployments and provider interactions with alert messages and prompts will be tracked over time following USAP corporate management and communications standards to measure engagement with the intervention. Draft peer comparison scripts alerts appear in **Appendix 1**; final wording of peer comparison scripts will be determined by USAP executive leadership.

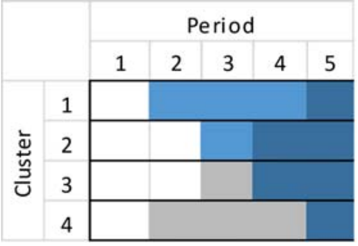

Figure. 2 x 2 factorial stepped wedge design.[1] Each cluster will be composed of 5-7 USAP divisions. In the initial 8-week period of the study, no clusters will receive either intervention; subsequently, clusters will be randomized at 8 week intervals to receive either intervention 1 (clinician peer comparisons, blue cells) or intervention 2 (patient letter, stippled cells), followed by a period were both interventions are simultaneously deployed at all

Letters will be distributed to eligible patients within the 2 weeks prior to surgery. Letters will be produced on USAP letterhead and signed by the USAP Chief Quality Officer (Dutton). Letter text will state USAP's commitment to brain health and avoiding potentially unnecessary medications for patients undergoing surgery. It will also include a statement encouraging patients to discuss anesthesia plans with their clinicians at the time of surgery to aid tailoring of care to individual needs. Letters will be distributed directly to patients via text link, email, or hard copy as a component of standard pre-operative instructional communications and other educational and patient outreach materials related to clinical care. USAP clinicians will be notified via e-mails from national and local leadership about the letter intervention, with information provided regarding letter content and goals. A draft letter appears in **Appendix 2**. Final wording of informational letter text will be determined by USAP executive leadership.

All study data will be obtained from the USAP clinical and quality data warehouse. This secure data warehouse contains billing, clinical, and survey response data routinely used by USAP for performance monitoring and quality improvement projects. Key data elements to be extracted from the USAP data warehouse for the present study will include: (1) baseline patient and surgery characteristics, benzodiazepine administration, complications, and length of stay, which come from anesthesia record data routinely obtained by USAP on all cases for billing purposes and from EMR data used by USAP for

quality monitoring in selected practices; and (2) patient demographics (race, ethnicity) and APSQ2 responses, which are collected from all USAP patients routinely after surgery via a secure web-based data collection platform (SurveyVitals, Inc., Springtown, TX). Finally, we will measure engagement of clinicians and patients with the study interventions via tracking of app and patient messaging engagements. We will query patients at the time of APSQ2 data collection to confirm discussions of letter content with the anesthesia team on the day of surgery. Study data will be sent securely to the University of Pennsylvania for analysis on secure, encrypted server under USAP's standard data sharing policy.

9. Analysis plan

Initial analyses will use descriptive statistics to examine the distribution of study variables overall and to compare baseline patient and clinician data across the control versus intervention units. All analyses will be via intention-to-treat, such that observations within randomization units will be analyzed according to the treatment assigned via randomization. We will use a mixed effects logistic regression to estimate the treatment effect of peer comparisons, informational letters for patients, or both versus control on the odds of receiving a benzodiazepine during anesthesia care. This model will contain binary indicator variables for the treatment status of each unit within a given period for each of the 2 main study interventions and an interaction term capturing the joint effect of the interventions. As care patterns may be similar across USAP divisions, we will include a random effect for the division. Time (study month) will be included as a fixed effect;[34] interactions to capture time-cluster and time-treatment effect heterogeneity will be considered in supplemental analyses.[35] Standard errors will be adjusted for heteroscedasticity[36] and clustering using standard methods.[37] The magnitude and significance of the coefficients on the main effects terms for each treatment and their interaction in the model will be interpreted as the independent and joint association of the study interventions on the primary outcome.

We will use a linear mixed effects model to estimate the independent and joint effects of the study interventions on the continuous APSQ2 patient satisfaction score, using the same modeling approach as described above. Similar mixed effects logistic or linear models will be used as appropriate based on outcome variable distributions to predict (1) delirium; (2) postoperative pulmonary complications; and (3) length of stay.[38] We will assess for heterogeneity of treatment effects across subgroups as defined above by adding interaction terms to main study models as appropriate.[39] Missing data rates across divisions and arms will be compared for all endpoints and patterns of missingness evaluated. Where missing outcome data rates are substantial (>10%), sensitivity analysis will be conducted using inverse probability weighting[40] to model the potential impact of missing data on study findings.

Analyses will assess heterogeneity of treatment effects across defined patient and provider subgroups in which we hypothesize that the interventions may have differing impacts; these will include: (1) patients aged 85 years and older versus others; (2) sex as recorded in the medical record; (3) race and ethnicity as obtained by patient report and from the EMR (where available), to be categorized based on initial data analyses; (3) insurance status; (4) procedure type; (5) patients treated by providers with higher vs lower historical rates of benzodiazepine administration.

Assuming a baseline benzodiazepine use rate of 45%, a total sample size of 225,000 at 24 divisions over 5 periods will provide over 90% power to detect a 2 percentage point change in the primary outcome for each intervention alone, and a 4 percentage point change for the combined intervention, at a Bonferroni-adjusted significance level of 0.017 and a conservative intra-center correlation coefficient of 0.3.[41] Comparable power is anticipated for satisfaction analyses, even after allowing for non-response rates of 15-20%. We anticipate that data on in-hospital medical outcomes will be available for approximately 11,250 patients treated at 4 USAP divisions. Assuming postoperative delirium and postoperative pulmonary complications each individually occur in 5% of control patients,[42-44] we will have 80% power to detect a 2.5 percentage point risk reduction for each outcome; assuming a more conservative 2%

control outcome rate,[38,42,45] we will have 80% power to detect a 1.5 percentage point risk reduction for each outcome, at a significance level of 0.017 and an intra-center correlation coefficient of 0.1.

Human research protection

10.1 Data confidentiality

Data on clinicians, patients, surgical procedures, and outcomes (benzodiazepine administration, patient satisfaction, complications, length of stay) will come from the USAP clinical and quality data warehouse. All data are proprietary to USAP and will be obtained for analysis by Penn investigators under USAP's standard data sharing agreement. Preparation of data files will be carried out by staff within the USAP Quality Division under the oversight of the USAP Chief Quality Officer. Data will be transferred and then stored, managed, and analyzed on a secure, encrypted server behind the University of Pennsylvania Health System (UPHS) firewall. All study personnel that will use this data are listed on the IRB application and have completed training in HIPAA standards and the CITI human subjects research. Data access will be password protected. Whenever possible, data will be de-identified for analysis.

Computer-based files will only be made available to personnel involved in the study through the use of access privileges and passwords. Wherever feasible, identifiers will be removed from study-related information. Precautions are already in place to ensure the data are secure by using passwords and HIPAA-compliant encryption.

10.2 Subject confidentiality

Data on clinicians and patients will be obtained from USAP staff files and the USAP clinical and quality data warehouse. Any information that is obtained will be used for research purposes only. Information on patients will only be disclosed within the study team. The clinician feedback messages will only provide aggregate numbers of cases with benzodiazepine administration and no individual patient information. All study staff will be reminded of the confidential nature of the data collected and contained in these databases. All study personnel that will use this data are listed on the IRB application and have completed training in HIPAA standards and the CITI human subjects research. Data access will be password protected. Whenever possible, data will be de-identified for analysis.

10.3 Subject privacy

All efforts will be made by study staff to ensure subject privacy. Data will be evaluated in a de-identified manner whenever possible.

10.4 Data disclosure

Information on clinicians and patients will not be disclosed to anyone outside of the study team.

10.5 Data safety and monitoring

The investigators from both USAP and University of Pennsylvania will provide oversight for the study evaluation of this corporate quality improvement initiative. Clinician practices will follow their standards of care to manage patients before, during, and after surgery.

10.6 Risk/benefit

10.6.1 Potential study risks

The potential risks associated with this study are minimal given the research is focused on an evaluation of a corporate quality improvement initiative. Breach of data is a potential risk that will be mitigated by using HIPAA compliant and secure data platforms for analysis a previously described.

10.6.2 Potential study benefits

The main potential benefit is knowledge gained on approaches that could reduce unnecessary benzodiazepine administration. Patients may benefit from less exposure to unnecessary benzodiazepines as a result. However, it is possible that patients will receive no benefit from this study.

10.6.3 Risk/benefit assessment

The risk/benefit ratio is favorable given the potential benefit of scientific knowledge that could be gained on how to change clinician and patient behavior to reduce unnecessary benzodiazepine administration. Efforts have been put into place to minimize the risk of breach of data. If favorable outcomes are found, then there is a potential to broadly disseminate findings to other physician practice groups.

References

1. Lyons VH, Li L, Hughes JP, Rowhani-Rahbar A. Proposed variations of the stepped-wedge design can be used to accommodate multiple interventions. J Clin Epidemiol 2017;86:160-167.
2. Griffin CE, 3rd, Kaye AM, Bueno FR, Kaye AD. Benzodiazepine pharmacology and central nervous system-mediated effects. Ochsner J 2013;13(2):214-23.
3. Cullen KA, Hall MJ, Golosinskiy A. Ambulatory surgery in the United States, 2006. Natl Health Stat Report 2009(11):1-25.
4. Hall MJ, DeFrances CJ, Williams SN, Gololinsky A, Schwartzman A. National Hospital Discharge Survey: 2007 Summary. National Health Statistics Reports 2010(29).
5. Spence J, Belley-Cote E, Devereaux PJ, et al. Benzodiazepine administration during adult cardiac surgery: a survey of current practice among Canadian anesthesiologists working in academic centres. Can J Anaesth 2018;65(3):263-271.
6. Weinstein SM, Poultsides L, Baaklini LR, et al. Postoperative delirium in total knee and hip arthroplasty patients: a study of perioperative modifiable risk factors. Br J Anaesth 2018;120(5):999-1008.
7. Lei VJ, Navathe AS, Seki SM, Neuman MD. Perioperative benzodiazepine administration among older surgical patients. Br J Anaesth 2021;127(2):e69-e71.
8. Maurice-Szamburski A, Auquier P, Viarre-Oreal V, et al. Effect of sedative premedication on patient experience after general anesthesia: a randomized clinical trial. JAMA 2015;313(9):916-25.
9. Boncyk C, Hess AS, Gaskell A, Sleigh J, Sanders RD. Does benzodiazepine administration affect patient satisfaction: a secondary analysis of the ConCIOUS study. Br J Anaesth 2017;118(2):266-267.
10. Marcantonio ER, Juarez G, Goldman L, et al. The relationship of postoperative delirium with psychoactive medications. JAMA 1994;272(19):1518-22.
11. Pandharipande PP, Pun BT, Herr DL, et al. Effect of sedation with dexmedetomidine vs lorazepam on acute brain dysfunction in mechanically ventilated patients: the MENDS randomized controlled trial. JAMA 2007;298(22):2644-53.
12. Riker RR, Shehabi Y, Bokesch PM, et al. Dexmedetomidine vs midazolam for sedation of critically ill patients: a randomized trial. JAMA 2009;301(5):489-99.
13. Sanders RD, Pandharipande PP, Davidson AJ, Ma D, Maze M. Anticipating and managing postoperative delirium and cognitive decline in adults. BMJ 2011;343:d4331.
14. Taipale PG, Ratner PA, Galdas PM, et al. The association between nurse-administered midazolam following cardiac surgery and incident delirium: an observational study. Int J Nurs Stud 2012;49(9):1064-73.
15. Whalin MK, Kreuzer M, Halenda KM, Garcia PS. Missed Opportunities for Intervention in a Patient With Prolonged Postoperative Delirium. Clin Ther 2015;37(12):2706-10.
16. Silva-Jr JM, Katayama HT, Nogueira FAM, Moura TB, Alves TL, de Oliveira BW. Comparison of dexmedetomidine and benzodiazepine for intraoperative sedation in elderly patients: a randomized clinical trial. Reg Anesth Pain Med 2019;44(3):319-324.
17. Cassel CK. Patient requests for nonbeneficial care. JAMA 2012;307(17):1797; author reply 1797-8.
18. Kerr EA, Kullgren JT, Saini SD. Choosing Wisely: How To Fulfill The Promise In The Next 5 Years. Health Aff (Millwood) 2017;36(11):2012-2018.
19. Niven DJ, Mrklas KJ, Holodinsky JK, et al. Towards understanding the de-adoption of low-value clinical practices: a scoping review. BMC Med 2015;13:255.

20. Bolder H. Identifying Medical Reversals: An Introduction to a New Area of Study NBER White Papers on the Value of Medical Research,. NBER. (https://www.nber.org/aging/valmed/WhitePaper-Bolder2.2019.pdf).
21. American Geriatrics Society abstracted clinical practice guideline for postoperative delirium in older adults. J Am Geriatr Soc 2015;63(1):142-50.
22. American Geriatrics Society 2019 Updated AGS Beers Criteria(R) for Potentially Inappropriate Medication Use in Older Adults. J Am Geriatr Soc 2019;67(4):674-694.
23. Hughes CG, Boncyk CS, Culley DJ, et al. American Society for Enhanced Recovery and Perioperative Quality Initiative Joint Consensus Statement on Postoperative Delirium Prevention. Anesth Analg 2020;130(6):1572-1590.
24. Thaler R, Sunstein C. Nudge: Improving Decisions about Health, Wealth, and Happiness. New Haven, CT: Yale University Press, 2008.
25. Harrison JD, Patel MS. Designing Nudges for Success in Health Care. AMA J Ethics 2020;22(9):E796-801.
26. Sedrak MS, Myers JS, Small DS, et al. Effect of a Price Transparency Intervention in the Electronic Health Record on Clinician Ordering of Inpatient Laboratory Tests: The PRICE Randomized Clinical Trial. JAMA Intern Med 2017;177(7):939-945.
27. Patel MS, Kurtzman GW, Kannan S, et al. Effect of an Automated Patient Dashboard Using Active Choice and Peer Comparison Performance Feedback to Physicians on Statin Prescribing: The PRESCRIBE Cluster Randomized Clinical Trial. JAMA Netw Open 2018;1(3):e180818.
28. Patel MS, Volpp KG, Asch DA. Nudge Units to Improve the Delivery of Health Care. N Engl J Med 2018;378(3):214-216.
29. Last BS, Buttenheim AM, Timon CE, Mitra N, Beidas RS. Systematic review of clinician-directed nudges in healthcare contexts. BMJ Open 2021;11(7):e048801.
30. Frank RG, Zeckhauser RJ. Custom-made versus ready-to-wear treatments: behavioral propensities in physicians' choices. J Health Econ 2007;26(6):1101-27.
31. Tannenbaum C, Martin P, Tamblyn R, Benedetti A, Ahmed S. Reduction of inappropriate benzodiazepine prescriptions among older adults through direct patient education: the EMPOWER cluster randomized trial. JAMA Intern Med 2014;174(6):890-8.
32. Martin P, Tamblyn R, Benedetti A, Ahmed S, Tannenbaum C. Effect of a Pharmacist-Led Educational Intervention on Inappropriate Medication Prescriptions in Older Adults: The D-PRESCRIBE Randomized Clinical Trial. JAMA 2019;320(18):1889-1898.
33. Lee TC, McDonald EG. Deprescribing Proton Pump Inhibitors: Overcoming Resistance. JAMA Intern Med 2020;180(4):571-573.
34. Hussey MA, Hughes JP. Design and analysis of stepped wedge cluster randomized trials. Contemp Clin Trials 2007;28(2):182-91.
35. Hemming K, Taljaard M, Forbes A. Analysis of cluster randomised stepped wedge trials with repeated cross-sectional samples. Trials 2017;18(1):101.
36. White H. A Heteroskedasticity-Consistent Covariance-Matrix Estimator and a Direct Test for Heteroskedasticity. Econometrica 1980;48(4):817-838.
37. Zeger SL, Liang KY. Longitudinal data analysis for discrete and continuous outcomes. Biometrics 1986;42(1):121-30.
38. Redelmeier DA, Thiruchelvam D, Daneman N. Delirium after elective surgery among elderly patients taking statins. CMAJ 2008;179(7):645-52.
39. Starks MA, Sanders GD, Coeytaux RR, et al. Assessing heterogeneity of treatment effect analyses in health-related cluster randomized trials: A systematic review. PLoS ONE 2019;14(8):e0219894.

40. Hogan JW, Lancaster T. Instrumental variables and inverse probability weighting for causal inference from longitudinal observational studies. Statistical Methods in Medical Research 2004;13(1):17-48.
41. Hemming K, Kasza J, Hooper R, Forbes A, Taljaard M. A tutorial on sample size calculation for multiple-period cluster randomized parallel, cross-over and stepped-wedge trials using the Shiny CRT Calculator. Int J Epidemiol 2020;49(3):979-995.
42. Inouye SK. Delirium in older persons. N Engl J Med 2006;354(11):1157-65.
43. Dasgupta M, Dumbrell AC. Preoperative risk assessment for delirium after noncardiac surgery: a systematic review. J Am Geriatr Soc 2006;54(10):1578-89.
44. Miskovic A, Lumb AB. Postoperative pulmonary complications. Br J Anaesth 2017;118(3):317-334.
45. Borzecki AM, Kaafarani HM, Utter GH, et al. How valid is the AHRQ Patient Safety Indicator "postoperative respiratory failure"? J Am Coll Surg 2011;212(6):935-45.

Appendix 1: Draft Peer Comparison Scripts

Top Performer Alert (top 10%)

You are a top performer. You have a low rate of benzodiazepine administration for older surgical patients undergoing general anesthesia.

You are a top performer this month.

- You are in the top 10% of providers.
- Based on your recent activity, you administered Midazolam to XX out of YY adults aged 65 or older who had general anesthesia.
- Please continue to work to limit potentially avoidable medications to older surgical patients.

Moderate Performer Alert (51%-11%)

Your benzodiazepine administration rate for older surgical patients undergoing general anesthesia is XX%. The top performer rate is YY%.

- Based on your recent activity, you administered Midazolam to XX out of YY adults aged 65 or older who had general anesthesia.
- You may be administering midazolam to too many older adults; this may contribute to postoperative cognitive dysfunction.
- ***Here*** is an evidence-based guideline on potentially avoidable medications in older adults [link to USAP educational materials]

Low Performer Alert (99%-50%)

Your benzodiazepine administration rate for older surgical patients undergoing general anesthesia is XX%. The median rate is YY%.

- Based on your recent activity, you administered Midazolam to XX out of YY adults aged 65 or older who had general anesthesia.
- Your performance falls below the median USAP performance.
- You may be administering midazolam to too many older adults; this may contribute to postoperative cognitive dysfunction.
- ***Here*** is an evidence-based guideline on potentially avoidable medications in older adults [link to USAP educational materials]

Appendix 2: Draft Informational Letter for Patients

US ANESTHESIA
PARTNERS

12222 Merit Tower
Dallas, TX 75251
972-715-5000
www.usap.com

February 1, 2022

Subject: USAP's commitment to brain health

Dear Patient:

Thank you for trusting US Anesthesia Partners with your upcoming medical procedure. We are looking forward to caring for you.

As your anesthesia team, USAP is committed to keeping you healthy during and after surgery; this includes following recommended practices to limit drowsiness and confusion after your operation.

We understand that many people have questions about their surgery and anesthesia. As the Chief Quality Officer of USAP I encourage you to ask about the anesthesia plan, including care you will receive before, during, and after surgery, when you see us before your procedure. This will help us find the best choice of medicines for you and your specific situation.

Sincerely,

Richard P. Dutton, MD MBA
Chief Quality Officer, USAP

Adjunct Professor of Anesthesiology
Texas A&M University College of Medicine

DROP-Benzo (De-adopting Routine Preoperative Benzodiazepines for Older Surgical Patients): a randomized trial of behavioral strategies to reduce unnecessary midazolam administration to older surgical patients

Study Protocol

Version 1.2

Date 06/26/2022

Outline

1. Abstract
2. Overall objectives
3. Aims
    3.1 Primary outcome
    3.2 Secondary outcomes
4. Background
5. Study design
    5.1 Design
    5.2 Study duration
    5.3 Target population
    5.4 Accrual
    5.5 Key inclusion criteria
    5.6 Key exclusion criteria
6. Subject recruitment
7. Subject compensation
8. Study procedures
    8.1 Consent
    8.2 Procedures
9. Analysis plan
10. Human research protection
    10.1 Data confidentiality
    10.2 Subject confidentiality
    10.3 Subject privacy
    10.4 Data disclosure
    10.5 Data safety and monitoring
    10.6 Risk/benefit
        10.6.1 Potential study risks
        10.6.2 Potential study benefits
        10.6.3 Risk/benefit assessment

1. Abstract

Over 21 million surgical procedures take place among adults aged 65 and older in the US each year, and most older surgical patients in the US now receive benzodiazepines (e.g., midazolam, lorazepam) during anesthesia care. This occurs despite recommendations to avoid these medications in older patients due to associated medical risks and lack of demonstrated benefit. In other words, routine benzodiazepine administration to older surgical patients is likely to represent low-value care that is a suitable target for de-adoption. In this study, we will evaluate a United States Anesthesia Partners (USAP, Dallas, TX) quality improvement initiative using peer comparison feedback to clinicians and/or mailed informational letters to patients as strategies to encourage physician de-adoption of routine preoperative benzodiazepine administration to older surgical patients. In partnership with USAP, this study will be conducted using randomization to evaluate its effect.

2. Overall objectives

The objective of this research study is to evaluate the effect of a corporate quality improvement initiative carried out by a private medical group practice aiming to change clinician preoperative benzodiazepine administration behaviors for older adults using clinician peer comparison feedback and mailed informational letters to patients.

3. Aims

3.1 Primary outcome

The primary outcome measure is the change in the rate of benzodiazepine administration among eligible surgical cases.

3.2 Secondary outcome

The secondary outcome measure is the change in patient satisfaction with care, as measured by Anesthesiologist Patient Satisfaction Questionnaire Composite Satisfaction Score, Version 2 (APSQ2).

3.3 Exploratory outcomes

We will explore several other outcomes of interest. These will include changes in dose and type of administered benzodiazepines; selected APSQ2 composite domain scores; 30-day delirium and pulmonary complications; and hospital length of stay for patients not discharged directly home after surgery.

4. Background

Benzodiazepines (e.g., midazolam, lorazepam) are sedative-hypnotic medications[2] that are frequently administered during surgical anesthesia care. Over 21 million major surgeries occur each year among US adults aged 65 and older,[3,4] and available data[5,6] indicate that over half of all older surgical patients now receive benzodiazepines during anesthesia care. For example, in a recent study of 24,683 patients aged 65 and older undergoing a range of non-cardiac surgeries at 4 hospitals in one US academic health system from 2014-2018, we found that 64.5% (95% CI: 64.0%, 64.9%) received a benzodiazepine during anesthesia; while the rate of benzodiazepine administration decreased with increasing age, 23.8% (95% CI: 21.8%, 25.8%) of individuals aged 85 and older still received a benzodiazepine.[7]

For patients undergoing general anesthesia (medically induced unconsciousness), benzodiazepines are most commonly administered as an adjunctive therapy delivered immediately before the induction of

anesthesia with the intent of reducing anxiety and improving patient experience. Nonetheless, a 2015 multicenter randomized study of 1,062 patients found comparable satisfaction scores after surgery among patients assigned to receive a benzodiazepine, placebo, or no premedication, with higher rates of residual sedation after surgery in the benzodiazepine group.[8] Similar findings were obtained in a subsequent observational study of 260 patients who received a benzodiazepine versus no premedication before general anesthesia.[9] Benzodiazepines carry dose-dependent risks for older surgical patients, including delirium[10-15] and respiratory depression.[16] Taken together, available evidence suggests that much benzodiazepine administration to older adults undergoing general anesthesia is unlikely to produce benefits and may cause harm; in other words, it represents low-value care[17,18] that is suitable for de-adoption.[19,20] Indeed, the American Geriatrics Society[21,22] and others[23] recommend that benzodiazepines be avoided in older surgical patients.

In this context, persistently high rates of benzodiazepine administration to older surgical patients nationally highlight a need for effective, scalable interventions to support de-adoption of this low-value practice. Behavioral economic nudges represent promising but largely unexplored strategies to reduce excessive benzodiazepine administration to surgical patients. "Nudges" are subtle changes to the framing of information that can influence behavior.[24,25] Clinician-facing nudges that incorporate peer comparisons can improve medication selection and prescribing[26-28] by countering common biases and heuristics[29] that can contribute to failures to appropriately tailor medication regimens based on individual patient characteristics.[30] Patient-facing nudges, such as simple informational letters represent an additional strategy to motivate practice change. In other contexts, simple informational letters have been shown to activating patients to participate in medical decisions.[31-33] In the present context, simple informational letters from practice leaders may prompt patients to initiate conversations with providers regarding care at the time of surgery, potentially influencing provider treatment decisions.

5. Study design

5.1 Design

This research study will analyze results from a USAP initiative that uses a four-arm factorial stepped-wedge cluster randomized trial. Both interventions (clinician peer comparisons and informational patient letters) will be concurrently rolled out across 24 physician practice divisions within USAP. Timing of intervention roll-out within each division will be randomly assigned to permit evaluation of the effect of each intervention individually and combined. The total intervention period will be approximately 9 months (40 weeks) in duration.

5.2 Study duration

The study is expected to take 2 years to conduct including planning (6 mo), the 9-month active phase of trial, a 1-month period for final data collection, and 8 months for analysis and dissemination of results.

5.3 Target populations

Anesthesia clinicians employed by USAP administering general anesthesia to patients aged 65 and older.

5.4 Accrual

This is an evaluation of a health system intervention of approximately 4,200 clinicians across 24 USAP physician practice divisions.

5.5 Key inclusion criteria

All anesthesia clinicians employed by USAP will be included in the evaluation.

In the research study analysis, patients will be included in the primary sample if they are at least aged 65 and undergo an elective (scheduled) surgical or endoscopic procedure under general anesthesia.

5.6 Key exclusion criteria

In the research study analysis, patients will be excluded if they are unscheduled (urgent/emergent) cases due to inability to reliably deliver study interventions to this group; if they did not receive general anesthesia; or if they received a nerve block procedure.

6. Subject recruitment

Since this is an evaluation of a corporate quality improvement intervention, clinicians and patients will not be recruited or enrolled individually but instead an analysis will be conducted based on anesthesia cases. We estimate that the sample will include approximately 4,200 clinicians and 225,000 patients.

7. Subject compensation

No compensation will be offered in this study.

8. Study procedures

8.1 Consent

A waiver of informed consent is requested from both clinicians and patients. This is a corporate quality improvement initiative that will be implemented with or without the proposed research study. The study is an evaluation of that implementation which has support from leadership at USAP. Therefore, clinicians and their patients will not be consented as this is the standard of practice within the context of the corporate quality improvement initiative. Without a waiver of the consent, the initiative would still be implemented by the health system, but the study would be infeasible. There are several additional reasons why we feel a waiver of consent should be granted. First, it is not feasible to consent every clinician and patient at the 24 practice divisions, which span 8 US states and serve hundreds of procedural care locations. Second, if clinicians or patients in the control group were consented, they would know they were being monitored and this could influence their behavior. This could potentially disrupt the design of the evaluation and make interpretation of the findings challenging. Third, clinicians are not being forced to administer or avoid benzodiazepines in a specific manner, and patients are not being forced to request or decline benzodiazepines. Instead, they are each being guided toward evidence-based care practices but maintain their ability to act as they feel is appropriate. This is no different than standard of care in which clinicians and patients each make decisions based on available information. Since the study will not involve collection of information whose provision would directly impacts on the safety or welfare of subjects, we do not plan to undertake additional communication with enrolled patients to provide additional pertinent information regarding their participation or study findings.

8.2 Procedures

Randomization procedures. The following procedure will be used at USAP to randomize divisions, informed by study team input. Each division will be a randomization unit. Each division will be a randomization unit. Divisions will be assigned to blocks of 4 divisions each. Division assignment into blocks will be guided by the probability of obtaining ICD 10 data for that division, anticipated case volume, and geographic locations. Within each block, each division will be randomized into one of the 4 predefined treatment sequences (Figure) using the fixed block randomization algorithm.

In the initial 8-week period of the study, no clusters will receive either intervention. During this baseline data collection phase, all USAP clinicians will be receive standard organization educational materials regarding preoperative medication use in older surgical patients, incorporating available best evidence on potentially avoidable medications in the elderly. Providers will receive communication during this phase notifying them of the quality improvement initiative and its components. Subsequently, clusters will be randomized at 8 week intervals to receive either peer comparison feedback or the patient informational letter, followed by a period were both interventions are simultaneously deployed at all divisions. The USAP Chief Quality Officer will be notified at the start of the study of each division's assigned treatment sequence and oversee implementation of the below study procedures according to this assignment. The randomization algorithm, code, and original sequence will be maintained on a secure server, accessible by the study data management team, and blinded to other study personnel.

Peer comparison feedback will use data on the number of eligible cases in which a benzodiazepine was administered. Providers will receive monthly alerts via USAP's smartphone-based practice management application or via USAP corporate e-mail or corporate text message presenting individual-level data on their benzodiazepine administration patterns versus other USAP providers, using data from the USAP clinical and quality data warehouse. Comparative data will be delivered using a 3-month rolling average as follows: (a) If clinician is above median: informed how their data compares to the median; (b) If clinician is below median but above 10th percentile: informed how their data compares to the 10th percentile; (c) If clinician is 10th percentile or below: informed of their data and commended for being a "high performer." Data will be compared to peer clinicians. Alert deployments and provider interactions with alert messages and prompts will be tracked over time following USAP corporate management and communications standards to measure engagement with the intervention. Draft peer comparison scripts alerts appear in **Appendix 1**; final wording of peer comparison scripts will be determined by USAP executive leadership.

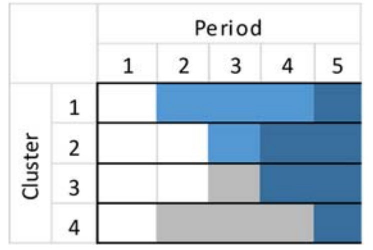

Figure. 2 x 2 factorial stepped wedge design.[1] Each cluster will be composed of 5-7 USAP divisions. In the initial 8-week period of the study, no clusters will receive either intervention; subsequently, clusters will be randomized at 8 week intervals to receive either intervention 1 (clinician peer comparisons, blue cells) or intervention 2 (patient letter, stippled cells), followed by a period were both interventions are simultaneously deployed at all

Letters will be distributed to eligible patients within the 2 weeks prior to surgery. Letters will be produced on USAP letterhead and signed by the USAP Chief Quality Officer (Dutton). Letter text will state USAP's commitment to brain health and avoiding potentially unnecessary medications for patients undergoing surgery. It will also include a statement encouraging patients to discuss anesthesia plans with their clinicians at the time of surgery to aid tailoring of care to individual needs. Letters will be distributed directly to patients via text link, email, or hard copy as a component of standard pre-operative instructional communications and other educational and patient outreach materials related to clinical care. USAP clinicians will be notified via e-mails from national and local leadership about the letter intervention, with information provided regarding letter content and goals. A draft letter appears in **Appendix 2**. Final wording of informational letter text will be determined by USAP executive leadership.

All study data will be obtained from the USAP clinical and quality data warehouse. This secure data warehouse contains billing, clinical, and survey response data routinely used by USAP for performance monitoring and quality improvement projects. Key data elements to be extracted from the USAP data warehouse for the present study will include: (1) baseline patient and surgery characteristics, benzodiazepine administration, complications, and length of stay, which come from anesthesia record data routinely obtained by USAP on all cases for billing purposes and from EMR data used by USAP for quality monitoring in selected practices; and (2) patient demographics (race, ethnicity) and APSQ2

responses, which are collected from all USAP patients routinely after surgery via a secure web-based data collection platform. Finally, we will measure engagement of clinicians and patients with the study interventions via tracking of app and patient messaging engagements. We will query patients at the time of APSQ2 data collection to confirm discussions of letter content with the anesthesia team on the day of surgery. Study data will be sent securely to the University of Pennsylvania for analysis on secure, encrypted server under USAP's standard data sharing policy.

9. Analysis plan

Initial analyses will use descriptive statistics to examine the distribution of study variables overall and to compare baseline patient and clinician data across the control versus intervention units. All analyses will be via intention-to-treat, such that observations within randomization units will be analyzed according to the treatment assigned via randomization. We will use a mixed effects logistic regression to estimate the treatment effect of peer comparisons, informational letters for patients, or both versus control on the odds of receiving a benzodiazepine during anesthesia care. This model will contain binary indicator variables for the treatment status of each unit within a given period for each of the 2 main study interventions and an interaction term capturing the joint effect of the interventions. As care patterns may be similar across USAP divisions, we will include a random effect for the division. Time (study month) will be included as a fixed effect;[34] interactions to capture time-cluster and time-treatment effect heterogeneity will be considered in supplemental analyses.[35] Standard errors will be adjusted for heteroscedasticity[36] and clustering using standard methods.[37] The magnitude and significance of the coefficients on the main effects terms for each treatment and their interaction in the model will be interpreted as the independent and joint association of the study interventions on the primary outcome.

We will use a linear mixed effects model to estimate the independent and joint effects of the study interventions on the continuous APSQ2 patient satisfaction score, using the same modeling approach as described above. Similar mixed effects logistic or linear models will be used as appropriate based on outcome variable distributions to predict (1) delirium; (2) postoperative pulmonary complications; and (3) length of stay.[38] We will assess for heterogeneity of treatment effects across subgroups as defined above by adding interaction terms to main study models as appropriate.[39] Missing data rates across divisions and arms will be compared for all endpoints and patterns of missingness evaluated. Where missing outcome data rates are substantial (>10%), sensitivity analysis will be conducted using inverse probability weighting[40] to model the potential impact of missing data on study findings.

Analyses will assess heterogeneity of treatment effects across defined patient and provider subgroups in which we hypothesize that the interventions may have differing impacts; these will include: (1) patients aged 85 years and older versus others; (2) sex as recorded in the medical record; (3) race and ethnicity as obtained by patient report and from the EMR (where available), to be categorized based on initial data analyses; (3) insurance status; (4) procedure type; (5) patients treated by providers with higher vs lower historical rates of benzodiazepine administration.

Assuming a baseline benzodiazepine use rate of 45%, a total sample size of 225,000 at 24 divisions over 5 periods will provide over 90% power to detect a 2 percentage point change in the primary outcome for each intervention alone, and a 4 percentage point change for the combined intervention, at a Bonferroni-adjusted significance level of 0.017 and a conservative intra-center correlation coefficient of 0.3.[41] Comparable power is anticipated for satisfaction analyses, even after allowing for non-response rates of 15-20%. We anticipate that data on in-hospital medical outcomes will be available for approximately 11,250 patients treated at 4 USAP divisions. Assuming postoperative delirium and postoperative pulmonary complications each individually occur in 5% of control patients,[42-44] we will have 80% power to detect a 2.5 percentage point risk reduction for each outcome; assuming a more conservative 2%

control outcome rate,[38,42,45] we will have 80% power to detect a 1.5 percentage point risk reduction for each outcome, at a significance level of 0.017 and an intra-center correlation coefficient of 0.1.

Human research protection

10.1 Data confidentiality

Data on clinicians, patients, surgical procedures, and outcomes (benzodiazepine administration, patient satisfaction, complications, length of stay) will come from the USAP clinical and quality data warehouse. All data are proprietary to USAP and will be obtained for analysis by Penn investigators under USAP's standard data sharing agreement. Preparation of data files will be carried out by staff within the USAP Quality Division under the oversight of the USAP Chief Quality Officer. Data will be transferred and then stored, managed, and analyzed on a secure, encrypted server behind the University of Pennsylvania Health System (UPHS) firewall. All study personnel that will use this data are listed on the IRB application and have completed training in HIPAA standards and the CITI human subjects research. Data access will be password protected. Whenever possible, data will be de-identified for analysis.

Computer-based files will only be made available to personnel involved in the study through the use of access privileges and passwords. Wherever feasible, identifiers will be removed from study-related information. Precautions are already in place to ensure the data are secure by using passwords and HIPAA-compliant encryption.

10.2 Subject confidentiality

Data on clinicians and patients will be obtained from USAP staff files and the USAP clinical and quality data warehouse. Any information that is obtained will be used for research purposes only. Information on patients will only be disclosed within the study team. The clinician feedback messages will only provide aggregate numbers of cases with benzodiazepine administration and no individual patient information. All study staff will be reminded of the confidential nature of the data collected and contained in these databases. All study personnel that will use this data are listed on the IRB application and have completed training in HIPAA standards and the CITI human subjects research. Data access will be password protected. Whenever possible, data will be de-identified for analysis.

10.3 Subject privacy

All efforts will be made by study staff to ensure subject privacy. Data will be evaluated in a de-identified manner whenever possible.

10.4 Data disclosure

Information on clinicians and patients will not be disclosed to anyone outside of the study team.

10.5 Data safety and monitoring

The investigators from both USAP and University of Pennsylvania will provide oversight for the study evaluation of this corporate quality improvement initiative. Clinician practices will follow their standards of care to manage patients before, during, and after surgery.

10.6 Risk/benefit

10.6.1 Potential study risks

The potential risks associated with this study are minimal given the research is focused on an evaluation of a corporate quality improvement initiative. Breach of data is a potential risk that will be mitigated by using HIPAA compliant and secure data platforms for analysis a previously described.

10.6.2 Potential study benefits

The main potential benefit is knowledge gained on approaches that could reduce unnecessary benzodiazepine administration. Patients may benefit from less exposure to unnecessary benzodiazepines as a result. However, it is possible that patients will receive no benefit from this study.

10.6.3 Risk/benefit assessment

The risk/benefit ratio is favorable given the potential benefit of scientific knowledge that could be gained on how to change clinician and patient behavior to reduce unnecessary benzodiazepine administration. Efforts have been put into place to minimize the risk of breach of data. If favorable outcomes are found, then there is a potential to broadly disseminate findings to other physician practice groups.

References

1. Lyons VH, Li L, Hughes JP, Rowhani-Rahbar A. Proposed variations of the stepped-wedge design can be used to accommodate multiple interventions. J Clin Epidemiol 2017;86:160-167.
2. Griffin CE, 3rd, Kaye AM, Bueno FR, Kaye AD. Benzodiazepine pharmacology and central nervous system-mediated effects. Ochsner J 2013;13(2):214-23.
3. Cullen KA, Hall MJ, Golosinskiy A. Ambulatory surgery in the United States, 2006. Natl Health Stat Report 2009(11):1-25.
4. Hall MJ, DeFrances CJ, Williams SN, Gololinsky A, Schwartzman A. National Hospital Discharge Survey: 2007 Summary. National Health Statistics Reports 2010(29).
5. Spence J, Belley-Cote E, Devereaux PJ, et al. Benzodiazepine administration during adult cardiac surgery: a survey of current practice among Canadian anesthesiologists working in academic centres. Can J Anaesth 2018;65(3):263-271.
6. Weinstein SM, Poultsides L, Baaklini LR, et al. Postoperative delirium in total knee and hip arthroplasty patients: a study of perioperative modifiable risk factors. Br J Anaesth 2018;120(5):999-1008.
7. Lei VJ, Navathe AS, Seki SM, Neuman MD. Perioperative benzodiazepine administration among older surgical patients. Br J Anaesth 2021;127(2):e69-e71.
8. Maurice-Szamburski A, Auquier P, Viarre-Oreal V, et al. Effect of sedative premedication on patient experience after general anesthesia: a randomized clinical trial. JAMA 2015;313(9):916-25.
9. Boncyk C, Hess AS, Gaskell A, Sleigh J, Sanders RD. Does benzodiazepine administration affect patient satisfaction: a secondary analysis of the ConCIOUS study. Br J Anaesth 2017;118(2):266-267.
10. Marcantonio ER, Juarez G, Goldman L, et al. The relationship of postoperative delirium with psychoactive medications. JAMA 1994;272(19):1518-22.
11. Pandharipande PP, Pun BT, Herr DL, et al. Effect of sedation with dexmedetomidine vs lorazepam on acute brain dysfunction in mechanically ventilated patients: the MENDS randomized controlled trial. JAMA 2007;298(22):2644-53.
12. Riker RR, Shehabi Y, Bokesch PM, et al. Dexmedetomidine vs midazolam for sedation of critically ill patients: a randomized trial. JAMA 2009;301(5):489-99.
13. Sanders RD, Pandharipande PP, Davidson AJ, Ma D, Maze M. Anticipating and managing postoperative delirium and cognitive decline in adults. BMJ 2011;343:d4331.
14. Taipale PG, Ratner PA, Galdas PM, et al. The association between nurse-administered midazolam following cardiac surgery and incident delirium: an observational study. Int J Nurs Stud 2012;49(9):1064-73.
15. Whalin MK, Kreuzer M, Halenda KM, Garcia PS. Missed Opportunities for Intervention in a Patient With Prolonged Postoperative Delirium. Clin Ther 2015;37(12):2706-10.
16. Silva-Jr JM, Katayama HT, Nogueira FAM, Moura TB, Alves TL, de Oliveira BW. Comparison of dexmedetomidine and benzodiazepine for intraoperative sedation in elderly patients: a randomized clinical trial. Reg Anesth Pain Med 2019;44(3):319-324.
17. Cassel CK. Patient requests for nonbeneficial care. JAMA 2012;307(17):1797; author reply 1797-8.
18. Kerr EA, Kullgren JT, Saini SD. Choosing Wisely: How To Fulfill The Promise In The Next 5 Years. Health Aff (Millwood) 2017;36(11):2012-2018.
19. Niven DJ, Mrklas KJ, Holodinsky JK, et al. Towards understanding the de-adoption of low-value clinical practices: a scoping review. BMC Med 2015;13:255.

20. Bolder H. Identifying Medical Reversals: An Introduction to a New Area of Study NBER White Papers on the Value of Medical Research,. NBER. (https://www.nber.org/aging/valmed/WhitePaper-Bolder2.2019.pdf).
21. American Geriatrics Society abstracted clinical practice guideline for postoperative delirium in older adults. J Am Geriatr Soc 2015;63(1):142-50.
22. American Geriatrics Society 2019 Updated AGS Beers Criteria(R) for Potentially Inappropriate Medication Use in Older Adults. J Am Geriatr Soc 2019;67(4):674-694.
23. Hughes CG, Boncyk CS, Culley DJ, et al. American Society for Enhanced Recovery and Perioperative Quality Initiative Joint Consensus Statement on Postoperative Delirium Prevention. Anesth Analg 2020;130(6):1572-1590.
24. Thaler R, Sunstein C. Nudge: Improving Decisions about Health, Wealth, and Happiness. New Haven, CT: Yale University Press, 2008.
25. Harrison JD, Patel MS. Designing Nudges for Success in Health Care. AMA J Ethics 2020;22(9):E796-801.
26. Sedrak MS, Myers JS, Small DS, et al. Effect of a Price Transparency Intervention in the Electronic Health Record on Clinician Ordering of Inpatient Laboratory Tests: The PRICE Randomized Clinical Trial. JAMA Intern Med 2017;177(7):939-945.
27. Patel MS, Kurtzman GW, Kannan S, et al. Effect of an Automated Patient Dashboard Using Active Choice and Peer Comparison Performance Feedback to Physicians on Statin Prescribing: The PRESCRIBE Cluster Randomized Clinical Trial. JAMA Netw Open 2018;1(3):e180818.
28. Patel MS, Volpp KG, Asch DA. Nudge Units to Improve the Delivery of Health Care. N Engl J Med 2018;378(3):214-216.
29. Last BS, Buttenheim AM, Timon CE, Mitra N, Beidas RS. Systematic review of clinician-directed nudges in healthcare contexts. BMJ Open 2021;11(7):e048801.
30. Frank RG, Zeckhauser RJ. Custom-made versus ready-to-wear treatments: behavioral propensities in physicians' choices. J Health Econ 2007;26(6):1101-27.
31. Tannenbaum C, Martin P, Tamblyn R, Benedetti A, Ahmed S. Reduction of inappropriate benzodiazepine prescriptions among older adults through direct patient education: the EMPOWER cluster randomized trial. JAMA Intern Med 2014;174(6):890-8.
32. Martin P, Tamblyn R, Benedetti A, Ahmed S, Tannenbaum C. Effect of a Pharmacist-Led Educational Intervention on Inappropriate Medication Prescriptions in Older Adults: The D-PRESCRIBE Randomized Clinical Trial. JAMA 2019;320(18):1889-1898.
33. Lee TC, McDonald EG. Deprescribing Proton Pump Inhibitors: Overcoming Resistance. JAMA Intern Med 2020;180(4):571-573.
34. Hussey MA, Hughes JP. Design and analysis of stepped wedge cluster randomized trials. Contemp Clin Trials 2007;28(2):182-91.
35. Hemming K, Taljaard M, Forbes A. Analysis of cluster randomised stepped wedge trials with repeated cross-sectional samples. Trials 2017;18(1):101.
36. White H. A Heteroskedasticity-Consistent Covariance-Matrix Estimator and a Direct Test for Heteroskedasticity. Econometrica 1980;48(4):817-838.
37. Zeger SL, Liang KY. Longitudinal data analysis for discrete and continuous outcomes. Biometrics 1986;42(1):121-30.
38. Redelmeier DA, Thiruchelvam D, Daneman N. Delirium after elective surgery among elderly patients taking statins. CMAJ 2008;179(7):645-52.
39. Starks MA, Sanders GD, Coeytaux RR, et al. Assessing heterogeneity of treatment effect analyses in health-related cluster randomized trials: A systematic review. PLoS ONE 2019;14(8):e0219894.

40. Hogan JW, Lancaster T. Instrumental variables and inverse probability weighting for causal inference from longitudinal observational studies. Statistical Methods in Medical Research 2004;13(1):17-48.
41. Hemming K, Kasza J, Hooper R, Forbes A, Taljaard M. A tutorial on sample size calculation for multiple-period cluster randomized parallel, cross-over and stepped-wedge trials using the Shiny CRT Calculator. Int J Epidemiol 2020;49(3):979-995.
42. Inouye SK. Delirium in older persons. N Engl J Med 2006;354(11):1157-65.
43. Dasgupta M, Dumbrell AC. Preoperative risk assessment for delirium after noncardiac surgery: a systematic review. J Am Geriatr Soc 2006;54(10):1578-89.
44. Miskovic A, Lumb AB. Postoperative pulmonary complications. Br J Anaesth 2017;118(3):317-334.
45. Borzecki AM, Kaafarani HM, Utter GH, et al. How valid is the AHRQ Patient Safety Indicator "postoperative respiratory failure"? J Am Coll Surg 2011;212(6):935-45.

Appendix 1: Draft Peer Comparison Scripts

Top Performer Alert (top 10%)

You are a top performer. You have a low rate of benzodiazepine administration for older surgical patients undergoing general anesthesia.

You are a top performer this month.

- You are in the top 10% of providers.
- Based on your recent activity, you administered Midazolam to XX out of YY adults aged 65 or older who had general anesthesia.
- Please continue to work to limit potentially avoidable medications to older surgical patients.

Moderate Performer Alert (50%-11%)

Your benzodiazepine administration rate for older surgical patients undergoing general anesthesia is XX%. The top performer rate is YY%.

- Based on your recent activity, you administered Midazolam to XX out of YY adults aged 65 or older who had general anesthesia.
- You may be administering midazolam to too many older adults; this may contribute to postoperative cognitive dysfunction.
- ***Here*** is an evidence-based guideline on potentially avoidable medications in older adults [link to USAP educational materials]

Low Performer Alert (99%-51%)

Your benzodiazepine administration rate for older surgical patients undergoing general anesthesia is XX%. The median rate is YY%.

- Based on your recent activity, you administered Midazolam to XX out of YY adults aged 65 or older who had general anesthesia.
- Your performance falls below the median USAP performance.
- You may be administering midazolam to too many older adults; this may contribute to postoperative cognitive dysfunction.
- ***Here*** is an evidence-based guideline on potentially avoidable medications in older adults [link to USAP educational materials]

Appendix 2: Draft Informational Letter for Patients

US ANESTHESIA
PARTNERS

12222 Merit Tower
Dallas, TX 75251
972-715-5000
www.usap.com

February 1, 2022

Subject: USAP's commitment to brain health

Dear Patient:

Thank you for trusting US Anesthesia Partners with your upcoming medical procedure. We are looking forward to caring for you.

As your anesthesia team, USAP is committed to keeping you healthy during and after surgery; this includes following recommended practices to limit drowsiness and confusion after your operation.

We understand that many people have questions about their surgery and anesthesia. As the Chief Quality Officer of USAP I encourage you to ask about the anesthesia plan, including care you will receive before, during, and after surgery, when you see us before your procedure. This will help us find the best choice of medicines for you and your specific situation.

Sincerely,

Richard P. Dutton, MD MBA
Chief Quality Officer, USAP

Adjunct Professor of Anesthesiology
Texas A&M University College of Medicine

Protocol Title: DROP-Benzo (De-adopting Routine Preoperative Benzodiazepines for Older Surgical Patients): a randomized trial of behavioral strategies to reduce unnecessary midazolam administration to older surgical patients

Protocol Number: 850809
Principal Investigator: Mark D. Neuman

Modification Summary of Changes

Substantive (non-editorial) Protocol Changes

Revision Date	Protocol Version	Applicable Section(s)	Page No.	Summary of Changes	Rationale for Changes
**May 23, 2022**	1.1	Section 8	5	Revised language in Section 8 ("Procedures") regarding block randomization procedure.<br><br>Original text: *"The divisions that will contribute data on complications and length of stay will be in the same block. The remaining divisions will be separated into 5 other blocks of 4 divisions each to obtain similar characteristics within each block, guided by the k-means clustering algorithm. Within each block, each division will be randomized into one of the 4 predefined treatment sequences (Figure) using the fixed block randomization algorithm."*<br><br>Revised text: *"Each division will be a randomization unit. Divisions will be assigned to blocks of 4 divisions each. Division assignment into blocks will be guided*	Language revised for clarity and accuracy.

Revision Date	Protocol Version	Applicable Section(s)	Page No.	Summary of Changes	Rationale for Changes
				by the probability of obtaining ICD 10 data for that division, anticipated case volume, and geographic locations. Within each block, each division will be randomized into one of the 4 predefined treatment sequences (Figure) using the fixed block randomization algorithm."	
**May 23, 2022**	1.1	Section 8	6	Revised language in Section 8 ("Procedures") regarding communication approach with USAP employed clinicians:<br><br>Original text: *"Providers will receive monthly alerts via USAP's smartphone-based practice management application presenting individual-level data on their benzodiazepine administration patterns versus other USAP providers."*<br><br>Revised text: *"Providers will receive monthly alerts via USAP's smartphone-based practice management application or via corporate e-mail or corporate text message presenting individual-level data on their benzodiazepine administration patterns*	Modified to align with USAP's standard organizational approaches to communication with employed clinicians.

Revision Date	Protocol Version	Applicable Section(s)	Page No.	Summary of Changes	Rationale for Changes
				versus other USAP providers."	
**June 26, 2022**	1.2	Section 8	5	Updated language describing the timing of randomization assignment notifications.<br><br>Original text: *The USAP Chief Quality Officer will receive notification of each site's randomization status two weeks before the beginning of each step in the randomization sequence and oversee implementation of the below study procedures according to the randomization assignment.*<br><br>Revised text: *The USAP Chief Quality Officer will be notified at the start of the study of each division's assigned treatment sequence and oversee implementation of the below study procedures according to this assignment.*	Updated to accommodate logistical considerations involved in deployment of study interventions by USAP team members.
**June 26, 2022**	1.2	Section 8	6-7	Vendor name removed.<br><br>Original text: *Key data elements to be extracted from the USAP data warehouse for the present study will include…APSQ2 responses, which are collected from all USAP patients routinely after surgery via a secure web-*	Updated to accommodate potential changes over time in USAP vendor relationships.

Revision Date	Protocol Version	Applicable Section(s)	Page No.	Summary of Changes	Rationale for Changes
				based data collection platform (SurveyVitals, Inc., Springtown, TX).<br><br>Revised text: *Key data elements to be extracted from the USAP data warehouse for the present study will include…APSQ2 responses, which are collected from all USAP patients routinely after surgery via a secure web-based data collection platform.*	

DROP-Benzo (De-adopting Routine Preoperative Benzodiazepines for Older Surgical Patients): a randomized trial of behavioral strategies to reduce unnecessary midazolam administration to older surgical patients

Statistical Analysis Plan

Date 08/01/2023

1. Introduction

DROP-Benzo is a stepped-wedge cluster randomized clinical trial conducted across 24 divisions of United States Anesthesia Partners (USAP, Dallas, TX). The primary objective of this trial is to assess the effectiveness of two different interventions — peer comparison feedback for clinicians and mailing informational letters — and their combined impact on routine preoperative benzodiazepine administration in elderly surgical patients.

This study employed a 2 x 2 factorial stepped wedge design, divided into 4 clusters, each following a unique, predetermined intervention sequence. During the initial 8 weeks, no clusters received any interventions. At weeks 8 and 16, two more clusters were randomly assigned to receive either intervention 1 (clinician peer comparisons, marked with blue cells) or intervention 2 (patient letters, designated by stippled cells). At weeks 24 and 32, the remaining two clusters — those that initiated intervention at a later or earlier stage — began implementing both interventions simultaneously.

Each USAP division served as a randomization unit and was allocated to one of the 4 clusters. To ensure balanced divisional characteristics across clusters, these divisions were stratified into six blocks. Factors to determine the stratification included the likelihood of obtaining ICD 10 data, projected case volume, and geographical locations. Within each block, divisions were randomly assigned to one of the four predetermined sequences.

2. Quality control

Prior to the analysis, we will review the distributions of each variable to be included in the analysis, aggregated across USAP divisions, to identify any outlying values that may be inaccurate and should be checked. To the extent possible, inaccuracies will be resolved and the data updated with the correct values. Data that are clearly incorrect but cannot be corrected will be excluded from the analyses. Data that are unusual but not impossible, and cannot either be verified or corrected, will remain in the analysis.

Quantifiable inclusion and exclusion criteria will be validated. Enrolled patients are required to be at least aged 65 and undergo an elective surgical or endoscopic procedure under general anesthesia. Patients undergoing primary regional anesthesia will be excluded from the study.

3. Baseline data

Baseline demographic and clinical variables will be examined to evaluate general trends and determine whether there are any notable imbalances that may lead to further adjustments. Continuous variables will be summarized through standard measures of central tendency and spread including means, medians, standard deviations and interquartile ranges (IQRs). Frequency distributions will be calculated for categorical variables. The summary statistics will be presented among control groups, intervention 1 group, intervention 2 group, and the combined intervention group.

4. Analysis of the primary outcome

The primary analysis will be intention-to-treat (ITT) analysis, i.e., patients within randomization units will be analyzed according to the intervention assigned via randomization. The primary analysis will use complete data, i.e., all subjects with valid outcomes, and additional sensitivity analysis will be conducted to include subjects with missing outcomes. All tests will be performed at a conservative significance level of 0.017, adjusted for the multiplicity of 3 comparisons within each model by the Bonferroni method.

The primary outcome is the use of benzodiazepine administration during the eligible surgery. We will use a mixed effects logistic regression model to estimate the treatment effect of peer comparisons, informational letters for patients, or both versus control on the odds of receiving a benzodiazepine during anesthesia care, accounting for within-division correlation and stratification, and adjusted for time (study month), age, gender, American Society of Anesthesiologists Physical Status Classification, and type of surgery. This model will contain binary indicator variables for each patient, determined by the intervention status assigned to their corresponding division during the corresponding time window, and an interaction term capturing the joint effect of the two interventions. As patients within the same division are likely to share similar care teams and protocols, a random effect across divisions will be included. An additional random effect representing the shared factors across divisions within the same block strata will also be included. Interaction terms that capture time-division and time-intervention effect heterogeneity will be considered in supplementary analyses. The Robust Variance Estimator (RVE) will be used to control for heteroscedasticity (Huber, 1967; White, 1980) or clustering (Liang and Zeger, 1986), which may occur due to various social networks, information sharing, or other potential confounders.

5. Analysis of secondary outcome

The secondary outcome is patient satisfaction with care, as measured by Anesthesiologist Patient Satisfaction Questionnaire Composite Satisfaction Score, Version 2 (APSQ2). We will use a linear mixed effects model to estimate the independent and joint effects of the study interventions on the continuous APSQ2 patient satisfaction score, using the same fixed and random effects as for the primary outcome. Normalization transformation will be applied if necessary. We will assess for heterogeneity of treatment effects across subgroups as defined above by adding interaction terms to main study models as appropriate.

6. Analysis of exploratory outcomes

Where data are available, we will assess the independent and joint effects of two interventions on several exploratory outcomes, including changes in dose and type of administered benzodiazepines, selected APSQ2 composite domain scores, 30-day delirium and pulmonary complications, and hospital length of stay for patients not discharged directly home after surgery. Linear or logistic mixed effects model, with similar fixed and random effects as specified in Section 4, will be used.

7. Sensitivity analysis for missing data

Baseline characteristics of patients with missing primary outcome will be compared with the baseline characteristics of those included in the analysis to assess potential for bias based on these missing values.

The primary analysis will use complete data, excluding individuals without outcome data. To assess the potential for bias incurred by ignoring the missing data, we will apply inverse-probability-weighting (IPW) method (Wooldridge, 2010) under the assumption of missing at random (MAR). For IPW, each subject will be weighted in the mixed-effects model by the inverse probability of being a complete case. We will estimate the non-missing rate of the primary outcome using patients' baseline demographic and clinical information, time, surgery type, and divisions' characteristics. Missing values in the covariates used in the IPW will be imputed using multiple imputation by chained equations (MICE, Buuren and Groothuis-Oudshoorn, 2011), with plausible values drawn from a predictive distribution. At least 10 imputed datasets will be created and the interventional effects obtained from these datasets will be averaged for inference.

9. Subgroup analysis

We will conduct subgroup analyses to evaluate heterogeneity of treatment effects across pre-specified patient and provider subgroups. These include: (1) patients aged 85 years and above versus other age groups; (2) sex as documented in the medical record; (3) race and ethnicity as reported by the patient and extracted from available Electronic Medical Record (EMR), with common categories determined by preliminary data analyses; (3) insurance status; (4) surgery type; (5) patients treated by providers with historically higher vs. lower rates of benzodiazepine administration.

10. Sample size estimate

Assuming a baseline benzodiazepine use rate of 45%, a total sample size of 225,000 at 24 USAP divisions over 5 periods will provide over 90% power to detect a 2 percentage point change in the primary outcome for each intervention alone, and a 4 percentage point change for the combined intervention, at a Bonferroni-adjusted significance level of 0.017 and a conservative intra-center correlation coefficient of 0.3. Comparable power is anticipated for satisfaction outcome, even after allowing for non-response rates of 15-20%. We anticipate that in-hospital medical outcome data will be available for approximately 11,250 patients treated at 4 USAP divisions. Assuming postoperative delirium and postoperative pulmonary complications each individually occur in 5% of control patients, we will have 80% power to detect a 2.5 percentage point risk reduction for each outcome; assuming a more conservative 2% outcome rate in controls, we will still have 80% power to detect a 1.5 percentage point risk reduction for each outcome, at a significance level of 0.017 and an intra-division correlation coefficient of 0.1.

References:

1. Hussey MA, Hughes JP. Design and analysis of stepped wedge cluster randomized trials. Contemp Clin Trials. 2007;28(2):182–91.
2. Barker D, McElduff P, D'Este C, Campbell MJ. Stepped wedge cluster randomised trials: a review of the statistical methodology used and available. BMC Med Res Methodol. 2016 Jun 6;16:69. doi: 10.1186/s12874-016-0176-5. Review.
3. Matthews JNS, Forbes AB. Stepped wedge designs: insights from a design of experiments perspective. Stat Med. 2017 Oct 30;36(24):3772-3790.
4. Huber, PJ. The behavior of maximum likelihood estimates under nonstandard conditions. Proceedings of the Fifth Berkeley Symposium on Mathematical Statistics and Probability, 1967; 1(1), 221-233.
5. White H. A heteroskedasticity-consistent covariance matrix estimator and a direct test for heteroskedasticity. Econometrica 1980;48: 817– 838.
6. Liang, KY, Zeger, SL. Longitudinal data analysis using generalized linear models. Biometrika. 1986; 73(1), 13-22.
7. Wooldridge, JM. 2010. Econometric Analysis of Cross Section and Panel Data. 2nd edition. MIT press.
8. Gruber JS et al. Estimation of treatment efficacy with complier average causal effects (CACE) in a randomized stepped wedge trial. Am J Epidemiol. 2014;179(9):1134–42.
9. Tannenbaum C, Martin P, Tamblyn R, Benedetti A, Ahmed S. Reduction of inappropriate benzodiazepine prescriptions among older adults through direct patient education: the EMPOWER cluster randomized trial. JAMA Intern Med. Jun 2014;174(6):890-898.
10. Hemming K, Kasza J, Hooper R, Forbes A, Taljaard M. A tutorial on sample size calculation for multiple-period cluster randomized parallel, cross-over and stepped-wedge trials using the Shiny CRT Calculator. Int J Epidemiol. 2020;49(3):979-995.
11. Hooper R, Teerenstra S, de Hoop E, Eldridge S. Sample size calculation for stepped wedge and other longitudinal cluster randomised trials. Stat Med. 2016 Nov 20;35(26):4718-4728.
12. Girling AJ, Hemming K. Statistical efficiency and optimal design for stepped cluster studies under linear mixed effects models. Stat Med. 2016 Jun 15;35(13):2149-66.
13. Van Buuren, S, Groothuis-Oudshoorn, K. MICE: Multivariate Imputation by Chained Equations in R. Journal of Statistical Software, 2011;45(3), 1-67.
