# Supplementary material for: Strategies to Limit Benzodiazepine Use in Anesthesia for Older Adults: A Randomized Clinical Trial
Source: JAMA Netw Open. 2024 Oct 31;7(10):e2442207. doi: 10.1001/jamanetworkopen.2024.42207 (PMC11528310; doi:10.1001/jamanetworkopen.2024.42207)
Supplement: Supplement 2. — eTable 1. Study Divisions eFigure 1. Study Randomization Scheme eFigure 2. Physician Peer Comparison Message: High-Performer Alert eFigure 3. Physician Peer Comparison Message: Moderate-Performer Alert eFigure 4. Physician Peer Comparison Message: Low-Performer Alert eFigure 5. Patient Informational Mailing Text eTable 2. Race and Ethnicity Information for Study Participants Providing Data eTable 3. Standardized Differences for Individual Study Covariates Versus Control Group eMethods. Information on Mixed-Effects Logistic Model eTable 4. Simplified Regression Model Results for Primary Outcome eFigure 6. Subgroup Analyses eTable 5. Patient Satisfaction Item Responses eTable 6. Adverse Events by Study Group [file jamanetwopen-e2442207-s002.pdf]

Supplemental Online Content

Neuman MD, Feng R, Shukla A, et al. Benzodiazepine reduction in anesthesia care for older adults: a randomized clinical trial. *JAMA Netw Open*. 2024;7(10):e2442207. doi:10.1001/jamanetworkopen.2024.42207

eTable 1. Study Divisions

eFigure1. Study Randomization Scheme

eFigure 2. Physician Peer Comparison Message: High-Performer Alert

eFigure 3. Physician Peer Comparison Message: Moderate-Performer Alert

eFigure 4. Physician Peer Comparison Message: Low-Performer Alert

eFigure 5. Patient Informational Mailing Text

eTable 2. Race and Ethnicity Information for Study Participants Providing Data

eTable 3. Standardized Differences for Individual Study Covariates Versus Control Group

eMethods. Information on Mixed-Effects Logistic Model

eTable 4. Simplified Regression Model Results for Primary Outcome

eFigure 6. Subgroup Analyses

eTable 5. Patient Satisfaction Item Responses

eTable 6. Adverse Events by Study Group

This supplemental material has been provided by the authors to give readers additional information about their work.

eTable 1: Study divisions. "Facilities" include hospitals, outpatient surgery centers, and physician offices. At the time of study planning, we anticipated implementation of the interventions at 423 facilities across 24 practice divisions. Prior to study start, division 11 (4 facilities) and division 18 (4 facilities) de-integrated from the group practice. These divisions were not included in the study and no data were collected on patients treated at these locations.		
**N**	**Study division**	**Number of facilities included**
**1**	**USAP Austin**	26
**2**	**USAP Colorado A**	10
**3**	**USAP Colorado B**	14
**4**	**USAP Colorado C**	27
**5**	**USAP Dallas A**	28
**6**	**USAP Dallas B**	21
**7**	**USAP Dallas C**	10
**8**	**USAP Dallas D**	55
**9**	**USAP Houston A**	8
**10**	**USAP Houston B**	16
**11**	**USAP Houston C**	4 [a]
**12**	**USAP Houston D**	26
**13**	**USAP Kansas**	2
**14**	**USAP Maryland A**	13
**15**	**USAP Maryland B**	40
**16**	**USAP Nevada A**	7
**17**	**USAP Nevada A**	26
**18**	**USAP Oklahoma**	4 [a]
**19**	**USAP Florida A**	30
**20**	**USAP Florida B**	7
**21**	**USAP Florida C**	13
**22**	**USAP San Antonio A**	11
**23**	**USAP San Antonio B**	11
**24**	**USAP Washington**	14

a. Division de-integrated prior to study start; no data collected

© 2024 Neuman MD et al. *JAMA Network Open*

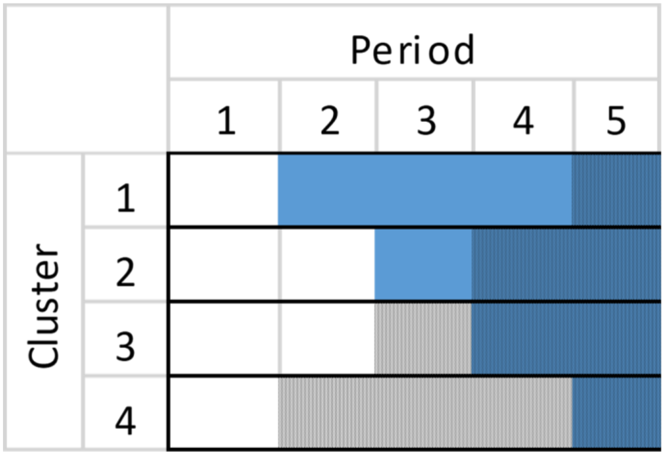

eFigure1. Study randomization scheme. Practices were randomized in clusters of 6 units to one of four study sequences shown above. In the initial period, no divisions received either intervention; each intervention (peer comparison feedback, blue cells; patient informational letter, stippled cells) was subsequently initiated at a new cluster of practices based on the assigned sequence.

MIDAZOLAM ADMINISTRATION SCORECARD

Quality Improvement Project Sponsored by USAP's Chief Quality Officer

Top Performer Alert!

You are a top performer. You have a low rate of benzodiazepine administration for older (>= 65 yrs) surgical patients undergoing general anesthesia.

Please continue to work to limit potentially avoidable medications to older surgical patients.

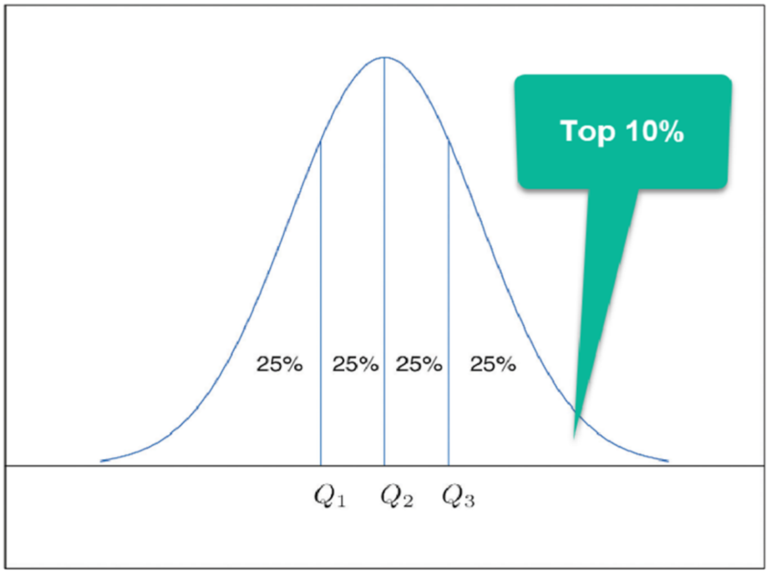

Questions? Email Richard.Dutton@usap.com

eFigure 2: Physician peer comparison message: high-performer alert

© 2024 Neuman MD et al. *JAMA Network Open*

MIDAZOLAM ADMINISTRATION SCORECARD

Quality Improvement Project Sponsored by USAP's Chief Quality Officer

Moderate Performer Alert!

Your benzodiazepine administration rate for older (>= 65 yrs) surgical patients undergoing general anesthesia falls between 11-51%. Top performer rate is <= 10%

You may be admnistering midazolam to too many older adults; this is known to contribute postoperative cognitive dysfunction.

Please continue to work to limit potentially avoidable medications to older surgical patients.

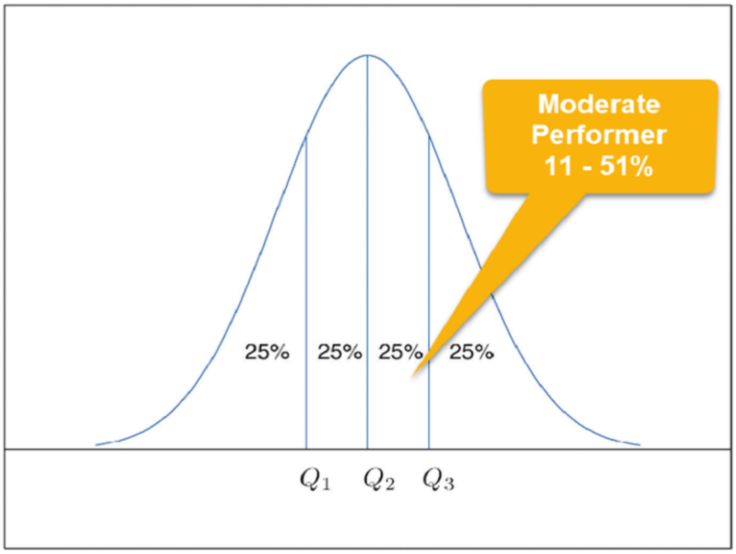

Questions? Email Richard.Dutton@usap.com

eFigure 3: Physician peer comparison message: moderate-performer alert

© 2024 Neuman MD et al. *JAMA Network Open*

MIDAZOLAM ADMINISTRATION SCORECARD

US Anesthesia Partners

Quality Improvement Project Sponsored by USAP's Chief Quality Officer

Opportunity for Improvement Alert!

Your benzodiazepine administration rate for older (>= 65 yrs) surgical patients undergoing general anesthesia is greater than 51%, which falls below the median USAP performance.

You may be admnistering midazolam to too many older adults; this is known to contribute postoperative cognitive dysfunction.

Please continue to work to limit potentially avoidable medications to older surgical patients.

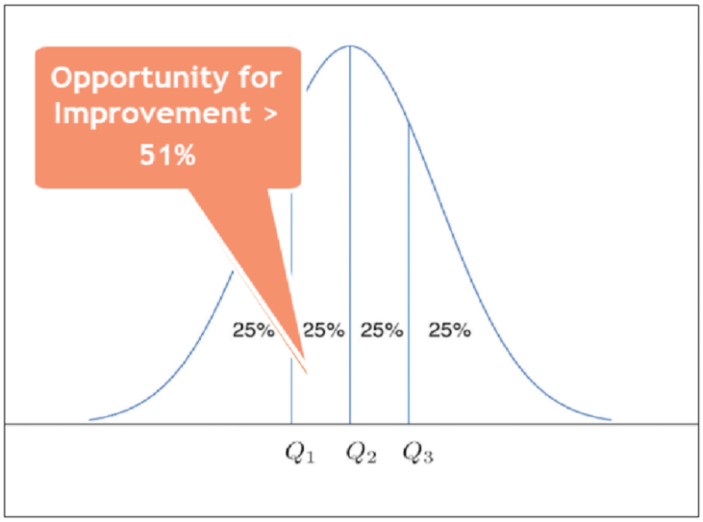

Questions? Email Richard.Dutton@usap.com

eFigure 4: Physician peer comparison message: low-performer alert

© 2024 Neuman MD et al. *JAMA Network Open*

Dear Patient:

Thank you for trusting US Anesthesia Partners with your upcoming medical procedure. We look forward to caring for you.

As your anesthesia team, USAP is committed to keeping you healthy during and after surgery. We understand that many people have questions about their anesthesia care, as well as the potential implications of anesthesia for brain health.

As the Chief Quality Officer of USAP, I encourage you to ask about the anesthesia plan--including care you will receive before, during, and after surgery--when you see us before your procedure. This will help us find the best choice of medicines for you and your specific situation.

Sincerely,

Richard P. Dutton, MD, MBA
Chief Quality Officer, USAP

Adjunct Professor of Anesthesiology
Texas A&M University College of Medicine

eFigure 5: Patient informational mailing text

© 2024 Neuman MD et al. *JAMA Network Open*

eTable 2: Race and ethnicity information for study participants providing data				
	**No intervention**	**Peer comparisons only**	**Patient mailing only**	**Peer comparisons + patient mailing**
**Race**				
White	11,858 (88.3%)	2,314 (88.3%)	2,581 (89.1%)	2,721 (88.1%)
Black or African American	917 (6.8%)	156 (6.0%)	201 (6.9%)	220 (7.1%)
American Indian or Alaskan Native	151 (1.1%)	17 (0.7%)	12 (0.4%)	13 (0.4%)
Asian	318 (2.4%)	46 (1.8%)	37 (1.6%)	48 (1.6%)
Other	136 (1.0%)	58 (2.2%)	39 (1.6%)	49 (1.6%)
More than one race	49 (0.36%)	30 (1.14%)	28 (1.3%)	39 (1.3%)
Total reporting	13,429	2,621	2,898	3,090
**Hispanic ethnicity**				
Yes	984 (7.2%)	153 (5.9%)	196 (6.7%)	230 (7.5%)
No	12,725 (92.8%)	2,441 (94.1%)	2,709 (93.2%)	2,857 (92.5%)
Total reporting	13,709	2,594	2,905	3,087

© 2024 Neuman MD et al. *JAMA Network Open*

eTable 3: Standardized differences for individual study covariates versus control group				
**Characteristic**		**Peer comparisons only**	**Patient mailing only**	**Peer comparisons + patient mailing**
Age in years		0.071	0.044	0.087
Male sex		0.014	-0.008	0.008
Female sex		-0.014	0.008	-0.008
Case duration in minutes		0.006	-0.061	0.0003
American Society of Anesthesiologists Physical Status				
	I: No systemic disease	0.005	-0.014	-0.014
	II: Mild systemic disease	-0.034	-0.003	-0.051
	III: Severe systemic disease	0.004	0.030	0.038
	IV or V: Severe systemic disease that is a constant threat to life or moribund patient	0.037	-0.037	0.016
Surgery type				
	Endoscopic	-0.030	0.025	-0.022
	General/abdominal	-0.031	-0.040	-0.017
	Orthopedic	0.051	0.046	0.021
	Urologic	-0.015	-0.098	-0.009
	Neurosurgery/Spine	-0.025	0.043	-0.012
	Cardiology	0.039	0.030	0.007
	Vascular	0.017	-0.002	0.007
	Cardiac	0.016	0.009	-0.001
	Ear, nose, and throat	-0.006	-0.033	-0.004
	Gynecology	-0.038	-0.005	-0.022
	Thoracic	0.011	-0.014	0.019
	Ophthalmology	0.012	0.002	-0.015
	Cosmetic	-0.013	-0.046	-0.015

© 2024 Neuman MD et al. *JAMA Network Open*

eTable 3: Standardized differences for individual study covariates versus control group				
**Characteristic**		**Peer comparisons only**	**Patient mailing only**	**Peer comparisons + patient mailing**
	Radiology	0.007	-0.017	-0.010
	Other	0.051	0.055	0.091
Insurance type				
	Commercial	0.004	0.026	0.013
	Government	-0.001	-0.013	-0.006
	Other	-0.006	-0.030	-0.017
Years in practice		0.031	0.055	-0.034
Care mode				
	Solo	0.083	0.021	-0.139
	Team	-0.083	-0.021	0.139
Service location				
	Inpatient hospital/Emergency Department	-0.081	0.056	-0.011
	Other	0.081	-0.056	0.011
Race				
	White	0.000	0.025	-0.006
	Black or African American	-0.033	0.004	0.012
	American Indian or Alaskan Native	-0.042	-0.081	-0.081
	Asian	-0.042	-0.082	-0.057
	Other	0.095	0.028	0.053
	More than one race	0.090	0.078	0.104
Hispanic ethnicity				
	Yes	-0.052	-0.016	0.011
	No	0.052	0.016	-0.011

© 2024 Neuman MD et al. *JAMA Network Open*

Supplementary methods: information on mixed-effects logistic model

The mixed-effects logistic model can be written as:

$Logit(P(Y_{ijk}=1))= \beta_0 + \beta_1$* treatmentA+$\beta_2$* treatmentB+$\beta_3$* treatmentA & treatmentB + β_4* age + β_5* male + β_6* surgery duration + β_7* severe ASA (III or IV) + β_8* surgery type 1 +….+ β_{21}*surgery type 15 + +β_{22} * private insurance + β_{23}* period 2 + …. + β_{26}*period 5 + $\gamma_{1j} + \gamma^k_{2j}$

Where

- I is the index for subject, j is the index for assigned cluster for subject i, k index for the center within each cluster.
- β_0 is the fixed intercept
- β_1, β_2, and β_3, are the fixed treatment and interaction effects
- β_4, …., β_{26} are the fixed covariate effects of age, gender, ASA status, surgery type, and time period.
- γ_{1j} represents the random effect for the j-th cluster (e.g., cluster-specific intercept) and is assumed to follow normal distributions with mean zero and variances $\sigma^2_{1.}$
- γ^k_{2j} represents the random effect for the k-th hospital nested within the j-th cluster, and is assumed to follow normal distributions with mean zero and variances σ^2_{2j}.

© 2024 Neuman MD et al. *JAMA Network Open*

eTable 4: Simplified regression model results for primary outcome.

	**Odds Ratio, 95% Confidence Interval[a]**
**Physician peer comparisons only**	1.00 (0.96-1.04)
**Patient mailing only**	1.00 (0.96-1.04)
**Peer comparisons plus patient mailing**	1.09 (1.04-1.14)

a. Adjusted for study period only

© 2024 Neuman MD et al. *JAMA Network Open*

eFigure 6: Subgroup analyses

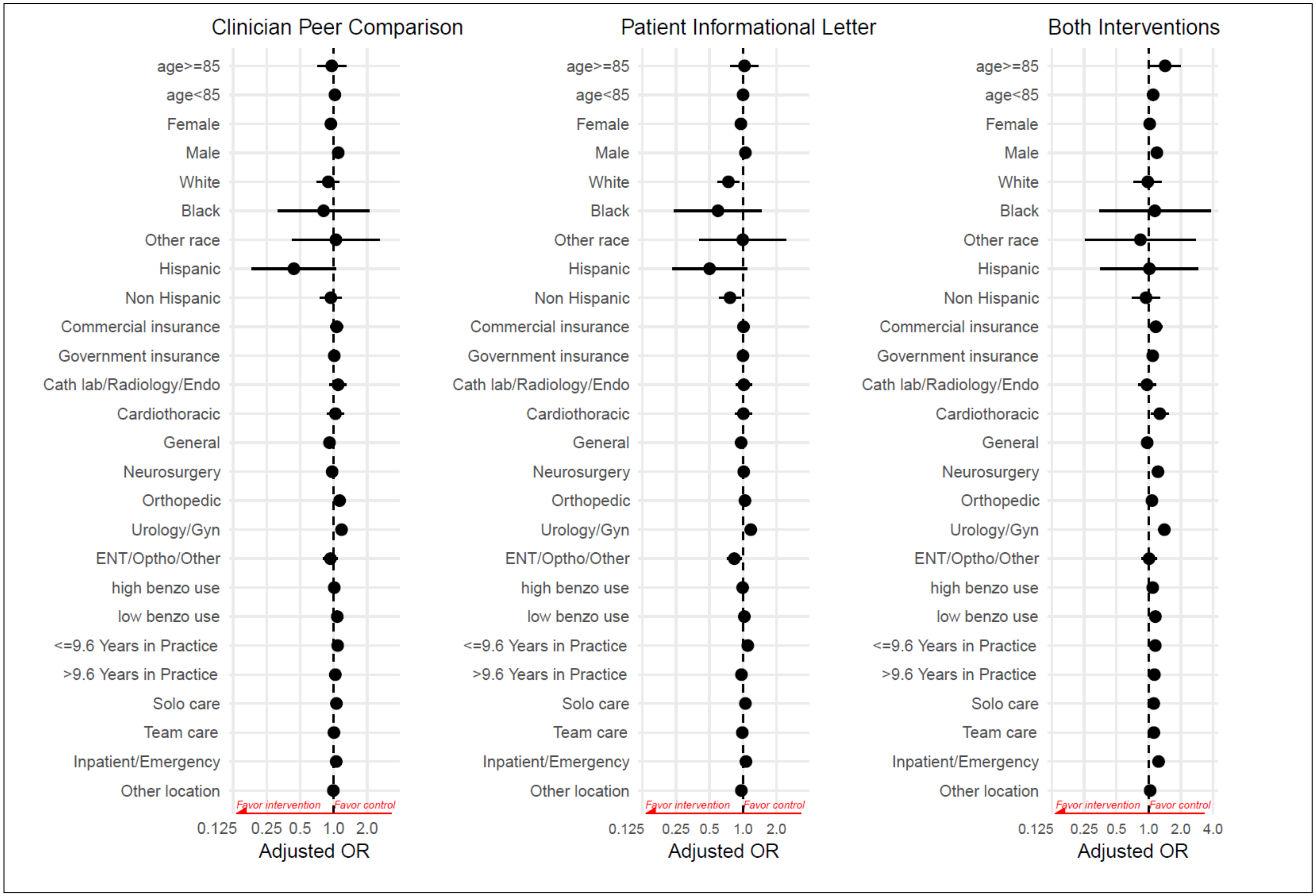

© 2024 Neuman MD et al. *JAMA Network Open*

eTable 5: Patient satisfaction item responses.					
		**No intervention (N=159674)**	**Intervention A only(N=81363)**	**Intervention B only(N=98520)**	**Intervention A+B(N=169712)**
Your options for anesthesia were explained before your surgery.	Disagree, disagree strongly, or neutral	1,576 (9.95%)	305 (5.77%)	349 (6.08%)	267 (5.67%)
	Agree or agree strongly	14,271 (90.05%)	4,983 (94.23%)	5,394 (93.92%)	4,439 (94.33%)
Your questions about anesthesia, the process, risks, and possible after effects were answered.	Disagree, disagree strongly, or neutral	1,127 (7.42%)	238 (4.98%)	224 (4.35%)	177 (4.14%)
	Agree or agree strongly	14,056 (92.58%)	4545 (95.02%)	4,920 (95.65%)	4,102 (95.86%)
You were well prepared to make informed decisions.	Disagree, disagree strongly, or neutral	878 (5.33%)	282 (5.11%)	327 (5.38%)	226 (4.45%)
	Agree or agree strongly	1,5596 (94.67%)	5,233 (94.89%)	5,754 (94.62%)	4,847 (95.55%)
Your anesthesia provider helped ease any anxiety you were feeling.	Disagree, disagree strongly, or neutral	936 (6.15%)	294 (5.51%)	302 (5.20%)	238 (4.99%)
	Agree or agree strongly	14,277 (93.85%)	5,039 (94.49%)	5,503 (94.80%)	4,531 (95.01%)

© 2024 Neuman MD et al. *JAMA Network Open*

eTable 5: Patient satisfaction item responses.

		No intervention (N=159674)	Intervention A only(N=81363)	Intervention B only(N=98520)	Intervention A+B(N=169712)
On a scale of 1 to 5, where 1 indicates the worst anesthesia experience and 5 indicates the best anesthesia experience, how would you rate your overall anesthesia experience?	Score 1, 2, or 3	812 (4.28%)	268 (4.00%)	308 (4.16%)	204 (3.61%)
	Score 4 or 5	18,144 (95.72%)	6,424 (96.00%)	7,098 (95.84%)	5,447 (96.39%)
Using a number from 5 to 1, where 5 is the best anesthesiologist possible and 1 is the worst, please rate your anesthesiologist.	Score 1, 2, or 3	462 (2.90%)	171 (3.03%)	167 (2.70%)	147 (2.91%)
	Score 4 or 5	15,495 (97.10%)	5,466 (96.97%)	6,011 (97.30%)	4,900 (97.09%)
Your anesthesia provider did his or her best to respect your privacy.	Disagree, disagree strongly, or neutral	527 (3.37%)	236 (4.33%)	263 (4.40%)	142 (2.88%)
	Agree or agree strongly	15,101 (96.63%)	5,211 (95.67%)	5,709 (95.60%)	4,794 (97.12%)
Your anesthesia provider ensured your comfort during the surgical experience.	Disagree, disagree strongly, or neutral	712 (4.36%)	240 (4.33%)	259 (4.26%)	178 (3.58%)
	Agree or agree strongly	15,633 (95.64%)	5,308 (95.67%)	5,827 (95.74%)	4,794 (96.42%)

© 2024 Neuman MD et al. *JAMA Network Open*

eTable 6: Adverse events by study group

Category	No intervention	Clinician peer comparison feedback	Patient mailing	Peer comparison + patient mailing
**Bleeding/transfusion**	5	4	5	2
**Cardiac complications**	178	110	93	163
**Death**	10	7	11	13
**Hyper/Hypoglycemia**	4	2	0	2
**Hyper/hypothermia**	34	7	10	29
**Injury/medication reaction/procedural complications**	35	15	14	34
**Neurological disorders**	13	10	6	5
**Other**	107	69	57	118
**Pain**	81	17	62	80
**Postoperative nausea/vomiting**	177	0	139	386
**Respiratory/airway**	158	89	76	132
**Vascular complications**	1	1	1	1
**Total**	803	331	474	965

© 2024 Neuman MD et al. *JAMA Network Open*
