# Supplementary material for: Strategies to Limit Benzodiazepine Use in Anesthesia for Older Adults: A Randomized Clinical Trial
Source: JAMA Netw Open. 2024 Oct 31;7(10):e2442207. doi: 10.1001/jamanetworkopen.2024.42207 (PMC11528310; doi:10.1001/jamanetworkopen.2024.42207)
Supplement: Supplement 3. — Data Sharing Statement [file jamanetwopen-e2442207-s003.pdf]

Data Sharing Statement

Neuman. Strategies to Limit Benzodiazepine Use in Anesthesia for Older Adults. *JAMA Netw Open*. Published October 31, 2024. doi:10.1001/jamanetworkopen.2024.42207

Data
Additional Information: Clinicaltrials.gov identifier NCT05436392
Data available: No

Additional Information
Explanation for why data not available: Unable to share data due to DUA terms
